# Fluoride as a Modifier of Metallome Homeostasis: A Systematic Review of Animal Studies

**DOI:** 10.1111/bcpt.70295

**Published:** 2026-09-04

**Authors:** Natalia Macedo‐Ribeiro, Jonas Tostes‐Figueiredo, Davi da Silva Barbirato, Isabel Maria Porto, Eliude Barbosa Gomes, Erico Marlon Moraes Flores, Marília Afonso Rabelo Buzalaf, Fernando Barbosa, Raquel Fernanda Gerlach

**Affiliations:** ^1^ Department of Basic and Oral Biology, Faculty of Dentistry of Ribeirao Preto University of Sao Paulo (FORP/USP) Ribeirao Preto Sao Paulo Brazil; ^2^ Department of Oral Surgery and Periodontology, Faculty of Dentistry of Ribeirao Preto University of Sao Paulo (FORP/USP) Ribeirao Preto Sao Paulo Brazil; ^3^ Department of Morphology and Children's Clinic, School of Dentistry at Araraquara Sao Paulo State University—UNESP (FOAr/UNESP) Araraquara Sao Paulo Brazil; ^4^ Department of Chemistry Federal University of Santa Maria Santa Maria Rio Grande do Sul Brazil; ^5^ Department of Biological Sciences, Bauru School of Dentistry University of São Paulo Bauru Sao Paulo Brazil; ^6^ Laboratory of Toxicology, School of Pharmaceutical Sciences of Ribeirao Preto University of Sao Paulo (FCFRP/USP) Ribeirao Preto Sao Paulo Brazil

**Keywords:** animal experimentation, fluorides, metallome, metals, trace elements

## Abstract

Fluoride is widely used for caries prevention due to its effects on mineralized tissues, yet its potential role as a modifier of systemic metal homeostasis remains insufficiently explored. This systematic review synthesizes preclinical evidence on the association between fluoride exposure and changes in metal and semi‐metal concentrations across biological matrices. A comprehensive search strategy was conducted across major databases without language or date restrictions, following SyRF, CAMARADES and PRISMA 2020 guidelines. Thirty‐one animal studies were included, encompassing multiple species, exposure conditions and analytical approaches. Despite substantial methodological heterogeneity, consistent patterns emerged. Fluoride exposure was associated with element‐specific redistribution of the metallome rather than uniform change. Essential elements were predominantly depleted, most consistently zinc, copper and manganese, whereas the toxic metals lead and cadmium tended to be retained. This contrast between homeostatically regulated essential elements that are lost and non‐regulated toxic metals that accumulate supports the hypothesis that fluoride differentially modifies the distribution and retention of co‐existing elements. The novelty of this review lies in integrating metallomic outcomes across experimental models, highlighting fluoride as a potential systemic modulator rather than a tissue‐specific agent. Although variability in study design and risk of bias limits causal inference, the consistent directionality of findings across models reinforces their biological plausibility and translational relevance.

## Plain Language Summary (PSL)

Fluoride helps prevent dental caries, but its effects are not limited to teeth and may also affect other organs and tissues. In this review, we examined animal studies to assess whether fluoride exposure changes how metals and similar elements are distributed and stored in the body. Across studies, fluoride was associated with changes in essential elements such as calcium, magnesium, zinc, copper and iron, as well as greater accumulation of toxic metals such as lead and cadmium in some tissues. These findings show that fluoride can alter elemental balance in the body and may contribute to functional impairment in specific organs and tissues.

## 1 Introduction

Fluoride has been widely used in dentistry due to its ability to enhance dental enamel remineralization and inhibit cariogenic processes, mainly by promoting the formation of fluorapatite (Ca_10_(PO_4_)_6_F_2_), which is less soluble under acidic conditions and more resistant to demineralization than hydroxyapatite (Ca_10_(PO_4_)_6_(OH)_2_), the principal inorganic constituent of the dental enamel [1]. This mechanism underlies its use in clinical practice [2, 3, 4, 5] and in community water fluoridation programmes worldwide, where exposure is controlled to balance efficacy and safety [6, 7, 8]. Despite these benefits, excessive fluoride exposure during critical periods of enamel development results in dental fluorosis, characterized by hypomineralization and structural enamel anomalies. The occurrence of fluorosis reflects the narrow therapeutic window of fluoride and underscores the importance of dose and timing in its biological effects [9].

Although traditionally associated with mineralized tissues, fluoride is a biologically active ion whose systemic effects extend beyond enamel. In this context, its potential role as a modulator of metal and semimetal homeostasis remains insufficiently explored.

From a chemical perspective, the hypothesis that fluoride may influence metallome homeostasis is biologically plausible and supported by well‐established principles of coordination chemistry. Fluoride is a hard Lewis base that interacts with a wide range of metal ions through complexation, ligand exchange, precipitation and dissolution reactions. As a consequence, fluoride can modify chemical equilibria involving metals, alter their speciation and influence the relative distribution of free and bound forms in aqueous systems. These principles are routinely discussed in classical inorganic, analytical, environmental and geochemical chemistry texts, where fluoride is recognized as an important ligand capable of affecting metal mobility, solubility and stability [10, 11, 12].

Although these concepts are well established in chemistry, their biological implications have received comparatively little attention. In biological systems, changes in metal speciation may influence absorption, transport, tissue retention, excretion, protein binding and participation in metal‐dependent biochemical processes. Therefore, fluoride should not be viewed solely as an ion that interacts with calcium phosphate minerals, but also as a potential modifier of the chemical equilibria governing the homeostasis of essential and toxic metals. This framework provides a mechanistic basis for interpreting reports of fluoride‐associated alterations in tissue concentrations of metals and semi‐metals observed in experimental studies.

In the present review, the term ‘metallome’ is used in an operational sense to refer to the tissue‐level profile of metals and metalloids, encompassing their concentrations, distribution and retention across biological matrices. This use is based on the definition proposed by Mounicou et al. [13], who described the metallome as the set of metals and metalloids present in a cell or tissue, including their identity, quantity and localization, and noted that it may be approached as a multielement concentration blueprint [13]. We therefore distinguish this use from stricter metallomics approaches focused on direct chemical speciation or metal–biomolecule complex characterization, and from the broader concept of the ionome, which encompasses all mineral nutrients and trace elements [14].

At the biochemical and cellular levels, fluoride ions (F^−^) are highly reactive and can interact with a variety of biological molecules and processes. Fluoride can inhibit certain enzymes [15, 16], disrupt organelle function [16, 17, 18], alter pH homeostasis [15, 16] and induce oxidative stress responses in cells [16, 17, 18, 19], effects that have been documented across in vitro and in vivo studies as part of the broader toxicological profile of this ion [20]. Beyond mineralized tissues, chronic or high‐level fluoride exposure has been associated with adverse systemic effects, including skeletal abnormalities (e.g., skeletal fluorosis) [9] and disturbances in soft tissues such as the liver and kidneys. The kidney has been repeatedly identified as a potential target organ, and experimental studies have reported oxidative stress, mitochondrial dysfunction and injury markers consistent with nephrotoxicity [17, 21, 22]. Evidence from experimental and critical toxicological reviews also supports the plausibility of soft‐tissue involvement, including hepatic alterations, although findings vary across models and exposure conditions [21].

Environmental and industrial contamination further complicate the landscape of fluoride exposure [23, 24, 25]. Fluoride occurs naturally in soil and water and is one of the more abundant elements in the earth's crust, often co‐occurring with other inorganic contaminants, including metals and semi‐metals [25, 26, 27]. Human activities such as mining, manufacturing and agricultural runoff have increased the prevalence of these elements in the environment, raising concerns about combined exposures and potential additive or synergistic toxic effects [28, 29].

In parallel, heavy metals and semi‐metals, including lead (Pb), cadmium (Cd) and arsenic (As), are environmentally persistent and bioaccumulative toxicants [29, 30] associated with a wide spectrum of adverse outcomes, such as neurotoxicity [31, 32, 33, 34], nephrotoxicity [35, 36, 37] and endocrine disruption [38, 39, 40]. Many of these effects arise from biochemical disruptions involving redox imbalance, mitochondrial dysfunction and altered cellular signalling, with well‐documented interference in metal‐dependent enzymatic processes [33, 35, 38]. Importantly, these toxic effects exhibit mechanistic overlap with fluoride‐induced biochemical disruptions, notably through shared oxidative stress pathways and disruption of metal‐dependent enzymes, providing strong biological plausibility for interactive effects under co‐exposure scenarios [15, 16].

In addition to studies directly assessing metal concentrations, a growing body of experimental evidence indicates that fluoride exposure induces broad molecular alterations consistent with disruptions in metal‐dependent biological processes. Proteomic and metabolic studies have shown that fluoride affects core aspects of cellular metabolism, particularly pathways related to energy production and mitochondrial function [41]. Under metabolically challenging conditions, fluoride has also been associated with enhanced oxidative stress and altered antioxidant defences, suggesting that its effects extend to cellular redox regulation and metabolic homeostasis [42]. Experimental findings further indicate that fluoride can trigger adaptive changes in mitochondrial activity, supporting the view that cellular responses to fluoride exposure are dynamic rather than uniform [43].

Consistent with this mechanistic plausibility, experimental studies have suggested potential interactions between fluoride and metals, particularly cadmium and lead, with co‐exposure sometimes resulting in additive or synergistic toxic effects on target organs such as the liver and kidneys. For example, animal studies have shown that combined exposure to fluoride and cadmium at individually non‐toxic doses exacerbates hepatic and renal injury via oxidative stress, mitochondrial dysfunction and inflammatory signalling compared with single‐agent exposure [44, 45]. Similarly, evidence from preclinical models indicates that fluoride exposure can modify lead toxicokinetics, increasing lead concentrations in blood and calcified tissues [46]. However, conclusions regarding these combined effects remain limited by substantial heterogeneity in experimental designs, species, exposure regimens and outcome measures, underscoring the need for a systematic synthesis and critical appraisal of the available preclinical evidence.

Despite the broad interest in fluoride's biological effects and the recognized toxicity of many metals, to our knowledge, there has been no comprehensive synthesis of preclinical laboratory evidence specifically addressing the association between fluoride exposure and changes in metal and semi‐metal concentrations across experimental models. Systematic evaluation of this evidence is critical to clarify whether fluoride exposure can influence the distribution, accumulation or biological behaviour of co‐existing inorganic elements in tissues, and to identify mechanistic patterns that might inform both toxicological understanding and future research priorities.

Given the biological plausibility of fluoride–metal interactions and the heterogeneity of preclinical findings, a comprehensive synthesis of experimental evidence is warranted. Therefore, the present study aims to examine, across preclinical laboratory models, whether fluoride exposure is associated with changes in the concentration, distribution and retention of metals and semi‐metals. Particular emphasis is placed on identifying consistent patterns across tissues and experimental conditions, as well as on evaluating the potential role of fluoride as a systemic modulator of metallome homeostasis.

## 2 Materials and Methods

This systematic review was designed to synthesize and critically appraise preclinical laboratory evidence on the association between fluoride exposure and changes in metal and semi‐metal concentrations, using established frameworks for preclinical evidence synthesis.

### 2.1 Protocol and Registration

This systematic review of preclinical laboratory evidence was conducted in accordance with the principles of the Systematic Review Facility (SyRF) (https://syrf.org.uk/projects) [47], which provides a structured framework for the transparent identification, selection, appraisal and synthesis of experimental studies, and followed the methodological recommendations of the Collaborative Approach to Meta‐Analysis and Review of Animal Experimental Studies (CAMARADES) (https://www.ed.ac.uk/clinical‐brain‐sciences/research/camarades/about‐camarades) for the critical appraisal of preclinical research. The conduct of the review emphasized independent and duplicate study selection, standardized data extraction and explicit assessment of internal validity and methodological rigour. The reporting of this review adheres to the Preferred Reporting Items for Systematic Reviews and Meta‐Analyses (PRISMA) 2020 statement [48], ensuring completeness, transparency and reproducibility of all methodological steps.

The review protocol was prospectively registered in the Open Science Framework (OSF; https://osf.io) under the associated project (Project DOI: 10.17605/OSF.IO/6AG4P; available at https://osf.io/kc3me). The full protocol is publicly accessible and includes detailed eligibility criteria, search strategies and planned methods for data synthesis.

No deviations from the registered protocol occurred during the conduct of the review.

### 2.2 Focused Question

The research question was structured according to the PICOD framework as follows: ‘What is the preclinical laboratory evidence regarding the association between fluoride exposure and changes in metal and semi‐metal concentrations in experimental models?’ This review was guided by a PICOD framework defined as follows:

- P—Population (experimental models): Pre‐clinical experimental models in the laboratory with animals exposed under controlled experimental conditions;
- I—Intervention/Exposure: Exposure to fluoride or fluoride‐containing compounds at any concentration, formulation or exposure duration applied experimentally;
- C—Comparator: Paired experimental conditions without fluoride exposure, baseline conditions, vehicle controls or alternative non‐fluoride exposures, when applicable;
- O—Outcomes*: Quantitative or qualitative changes in the concentrations of metals and semi‐metals assessed in biological, cellular or material matrices using validated analytical methods; and
- D—Design: Experimental in vivo laboratory studies designed to evaluate the effects of fluoride exposure under controlled conditions.

*The outcomes of interest were defined a priori and categorized as primary and secondary outcomes: (i) primary outcome: changes in the concentrations of metals and semi‐metals associated with experimental fluoride exposure, assessed qualitatively or quantitatively in biological, cellular or material matrices, as reported in preclinical laboratory studies; and (ii) secondary outcomes: (a) toxicological and biological effects—harmful biological consequences, adverse effects and indicators of systemic or cellular toxicity related to alterations in metal and semi‐metal concentrations following fluoride exposure, as reported in preclinical experimental models; (b) balance between beneficial and harmful effects—reported indicators allowing the appraisal of the balance between potential beneficial effects of fluoride exposure and associated harmful outcomes, when such information was available within individual preclinical studies; and (c) integrated qualitative assessment of evidence—a qualitative integration of preclinical evidence considering the balance between reported beneficial and harmful effects of fluoride exposure, methodological quality and risk of bias of included studies, and the presence or absence of experimental evidence supporting synergistic interactions between fluoride and metals or semi‐metals in bioaccumulation processes.

### 2.3 Study Selection Criteria

Studies were considered eligible if they met all of the following criteria:

- Preclinical experimental laboratory studies designed to evaluate the association between fluoride exposure and changes in the concentrations of metals and semi‐metals;
- Assessment of metal or semi‐metal concentrations in biological matrices, tissues, organs, cells or experimental substrates using quantitative or qualitative analytical methods; and
- Reporting of fluoride exposure under controlled experimental conditions, including information on concentration, formulation and exposure duration.

Studies were excluded if they met any of the following criteria:

- Studies in which the relationship between fluoride exposure and changes in metal or semi‐metal concentrations was indirect, unclear or not experimentally assessed;
- Studies lacking sufficient methodological detail on the fluoride intervention or on the assessment of metal or semi‐metal concentrations, precluding data extraction or critical appraisal; or
- Non‐experimental designs, narrative reports, reviews, conference abstracts or studies not conducted under controlled laboratory conditions.

No restrictions were applied regarding language or year of publication in the electronic searches.

### 2.4 Information Sources

A comprehensive electronic search was conducted across the following bibliographic databases: MEDLINE via PubMed (https://pubmed.ncbi.nlm.nih.gov), Cochrane Central Register of Controlled Trials (CENTRAL; https://www.cochranelibrary.com), Web of Science Core Collection (WoS; https://www.webofscience.com) accessed through Clarivate Analytics (https://clarivate.com), Embase (https://www.embase.com) and Scopus (https://www.scopus.com) through Elsevier (https://www.elsevier.com).

To reduce the risk of publication bias, grey literature sources were also searched, including the System for Information on Grey Literature in Europe (SIGLE) through the OpenGrey database (https://opengrey.eu), archived in the DANS EASY repository (Data Archiving and Networked Services).

Electronic searches were complemented by manual screening of reference lists from included studies and relevant specialty journals. Subject‐matter experts were identified via Expertscape (https://expertscape.com) and contacted to identify additional data sources. Only studies published in peer‐reviewed journals were eligible for inclusion.

### 2.5 Search Strategy

Search strategies were systematically developed and tailored for each database using controlled vocabulary terms, including Medical Subject Headings (MeSH), Emtree and database‐specific index terms, in addition to relevant free‐text keywords. Searches were conducted in PubMed, Cochrane CENTRAL, Web of Science, Embase, Scopus, grey literature sources and protocol registration databases. All search terms were combined using the Boolean operators ‘OR’ and ‘AND’. The complete search strategies for all databases, including exact search strings and applied limits, are provided in Table 1, in accordance with PRISMA 2020 recommendations [48].

**TABLE 1:** Search strategies.

Search strategy	Electronic databases
(‘in vitro techniques’[MeSH Terms] OR ‘in vitro’[Title/Abstract] OR ‘models, animal’[MeSH Terms] OR ‘animals, laboratory’[MeSH Terms] OR ‘animal experimentation’[MeSH Terms] OR ‘mice’[MeSH Terms] OR ‘rats’[MeSH Terms] OR ‘animal model’[Title/Abstract] OR ‘animal models’[Title/Abstract] OR ‘animals laboratory’[Title/Abstract] OR ‘laboratory animal’[Title/Abstract] OR ‘laboratory animals’[Title/Abstract] OR ‘animal experiments’[Title/Abstract] OR ‘animal experiment’[Title/Abstract] OR ‘mice’[Title/Abstract] OR ‘mus’[Title/Abstract] OR ‘mouse’[Title/Abstract] OR ‘rats’[Title/Abstract] OR ‘rat’[Title/Abstract]) AND (‘fluorides’[MeSH Terms] OR ‘fluorine compounds’[MeSH Terms] OR ‘fluorine’[MeSH Terms] OR ‘fluorides’[Title/Abstract] OR ‘fluorine’[Title/Abstract] OR ‘Fluoride’[Title/Abstract]) AND (‘metals’[MeSH Terms] OR ‘metals, heavy’[MeSH Terms] OR ‘heavy metal poisoning, nervous system’[MeSH Terms] OR ‘metalloids’[MeSH Terms] OR ‘metals, alkali’[MeSH Terms] OR ‘metals, alkaline earth’[MeSH Terms] OR ‘metals, light’[MeSH Terms] OR ‘metals, rare earth’[MeSH Terms] OR ‘trace elements’[MeSH Terms] OR ‘metals’[Title/Abstract] OR ‘metal’[Title/Abstract] OR ‘trace elements’[Title/Abstract] OR ‘trace element’[Title/Abstract] OR ‘biometal’[Title/Abstract])	MEDLINE|PubMed
ID Search Hits #1 MeSH descriptor: [in vitro techniques] explode all trees #2 MeSH descriptor: [in vitro] explode all trees #3 MeSH descriptor: [models, animal] explode all trees #4 MeSH descriptor: [animals, laboratory] explode all trees #5 MeSH descriptor: [animal experimentation] explode all trees #6 MeSH descriptor: [mice] explode all trees #7 MeSH descriptor: [rats] explode all trees #8 (animal model OR animal models OR animals, laboratory OR laboratory animal OR laboratory animals OR animal experiments OR animal experiment OR mice OR mus OR mouse OR rats OR Rat):ti,ab,kw #9 #1 OR #2 OR #3 OR #4 OR #5 OR #6 OR #7 OR #8 #10 MeSH descriptor: [fluorides] explode all trees #11 MeSH descriptor: [fluorine compounds] explode all trees #12 MeSH descriptor: [fluorine] explode all trees #13 (fluorides OR fluorine OR fluoride):ti,ab,kw #14 #10 OR #11 OR #12 OR #13 #15 MeSH descriptor: [metals] explode all trees #16 MeSH descriptor: [metals, heavy] explode all trees #17 MeSH descriptor: [metalloids] explode all trees #18 MeSH descriptor: [metals, alkali] explode all trees #19 MeSH descriptor: [heavy metal poisoning, nervous system] explode all trees #20 MeSH descriptor: [metals, alkaline earth] explode all trees #21 MeSH descriptor: [metals, light] explode all trees #22 MeSH descriptor: [metals, rare earth] explode all trees #23 MeSH descriptor: [trace elements] explode all trees #24 (metals OR metal OR trace elements OR trace element OR biometal):ti,ab,kw #25 #15 OR #16 OR #17 OR #18 OR #19 OR #20 OR #21 OR #22 OR #23 OR #24 #26 #9 AND #14 AND #25	Cochrane CENTRAL
((ALL = ((‘in vitro techniques’ OR ‘in vitro’ OR ‘models, animal’ OR ‘animals, laboratory’ OR ‘animal experimentation’ OR mice OR rats OR ‘animal model’ OR ‘animal models’ OR ‘animals, laboratory’ OR ‘laboratory animal’ OR ‘laboratory animals’ OR ‘animal experiments’ OR ‘animal experiment’ OR mice OR mus OR mouse OR rats OR rat))) AND ALL = ((fluorides OR ‘fluorine compounds’ OR fluorine OR fluorides OR fluorine OR fluoride))) AND ALL = ((metals OR ‘metals, heavy’ OR ‘heavy metal poisoning, nervous system’ OR metalloids OR ‘metals, alkali’ OR ‘metals, alkaline earth’ OR ‘metals, light’ OR ‘metals, rare earth’ OR ‘trace elements’ OR metals OR metal OR ‘trace elements’ OR ‘trace element’ OR biometals))	Web of Science
#1—‘in vitro techniques’/exp OR ‘in vitro techniques’ OR ‘in vitro’/exp OR ‘in vitro’ OR ‘models, animal’/exp OR ‘models, animal’ OR ‘animals, laboratory’/exp OR ‘animals, laboratory’ OR ‘animal experimentation’/exp OR ‘animal experimentation’ OR ‘mice’/exp OR ‘mice’ OR ‘rats’/exp OR ‘rats’ OR ‘animal model’:ti,ab,kw OR ‘animal models’:ti,ab,kw OR ‘animals, laboratory’:ti,ab,kw OR ‘laboratory animal’:ti,ab,kw OR ‘laboratory animals’:ti,ab,kw OR ‘animal experiments’:ti,ab,kw OR ‘animal experiment’:ti,ab,kw OR ‘mice’:ti,ab,kw OR ‘mus’:ti,ab,kw OR ‘mouse’:ti,ab,kw OR ‘rats’:ti,ab,kw OR ‘rat’:ti,ab,kw #2—‘fluorides’/exp OR ‘fluorides’ OR ‘fluorine compounds’/exp OR ‘fluorine compounds’ OR ‘fluorine’/exp OR ‘fluorine’ OR ‘fluorides’:ti,ab,kw OR ‘fluorine’:ti,ab,kw OR ‘fluoride’:ti,ab,kw #3—‘metals’/exp OR ‘metals’ OR ‘metals, heavy’/exp OR ‘metals, heavy’ OR ‘heavy metal poisoning, nervous system’/exp OR ‘heavy metal poisoning, nervous system’ OR ‘metalloids’/exp OR ‘metalloids’ OR ‘metals, alkali’/exp OR ‘metals, alkali’ OR ‘metals, alkaline earth’/exp OR ‘metals, alkaline earth’ OR ‘metals, light’/exp OR ‘metals, light’ OR ‘metals, rare earth’/exp OR ‘metals, rare earth’ OR ‘trace elements’/exp OR ‘trace elements’ OR ‘metals’:ti,ab,kw OR ‘metal’:ti,ab,kw OR ‘trace elements’:ti,ab,kw OR ‘trace element’:ti,ab,kw OR ‘biometal’:ti,ab,kw #1 AND #2 AND #3 AND (‘article’/it OR ‘article in press’/it OR ‘chapter’/it OR ‘conference paper’/it OR ‘conference review’/it OR ‘editorial’/it OR ‘erratum’/it OR ‘letter’/it OR ‘preprint’/it OR ‘review’/it OR ‘short survey’/it)	Embase
INDEXTERMS (‘in vitro techniques’ OR ‘in vitro’ OR ‘models, animal’ OR ‘animals, laboratory’ OR ‘animal experimentation’ OR mice OR rats) OR TITLE‐ABS‐KEY (‘animal model’ OR ‘animal models’ OR ‘animals, laboratory’ OR ‘laboratory animal’ OR ‘laboratory animals’ OR ‘animal experiments’ OR ‘animal experiment’ OR mice OR mus OR mouse OR rats OR rat) AND INDEXTERMS (fluorides OR ‘fluorine compounds’ OR fluorine) OR TITLE‐ABS‐KEY (fluorides OR fluorine OR fluoride) AND INDEXTERMS (metals OR ‘metals, heavy’ OR ‘heavy metal poisoning, nervous system’ OR metalloids OR ‘metals, alkali’ OR ‘metals, alkaline earth’ OR ‘metals, light’ OR ‘metals, rare earth’ OR ‘trace elements’) OR TITLE‐ABS‐KEY (metals OR metal OR ‘trace elements’ OR ‘trace element’ OR biometal)	Scopus
(‘in vitro techniques’ OR ‘in vitro’ OR ‘models, animal’ OR ‘animals, laboratory’ OR ‘animal experimentation’ OR mice OR rats OR ‘animal model’ OR ‘animal models’ OR ‘animals, laboratory’ OR ‘laboratory animal’ OR ‘laboratory animals’ OR ‘animal experiments’ OR ‘animal experiment’ OR mice OR mus OR mouse OR rats OR rat) AND (fluorides OR ‘fluorine compounds’ OR fluorine OR fluorides OR fluorine OR fluoride) AND (metals OR ‘metals, heavy’ OR ‘heavy metal poisoning, nervous system’ OR metalloids OR ‘metals, alkali’ OR ‘metals, alkaline earth’ OR ‘metals, light’ OR ‘metals, rare earth’ OR ‘trace elements’ OR metals OR metal OR ‘trace elements’ OR ‘trace element’ OR biometal)	Protocol registries and OpenGrey database

Electronic searches were initially completed on 30 October 2025, and subsequently updated on 20 January 2026. Automated alerts were established in all databases to identify newly published or ongoing studies up to the date of manuscript submission.

A comprehensive and reproducible search strategy was developed in accordance with the PRISMA‐S extension for reporting literature searches [49]. Electronic searches were conducted in major bibliographic databases relevant to biomedical and preclinical research.

To reduce the risk of publication bias, grey literature sources were also searched, including relevant thesis and dissertation repositories, conference proceedings and preprint servers, when applicable. Reference lists of included studies and relevant reviews were manually screened to identify additional eligible studies.

No restrictions on language or year of publication were applied. Full search strategies for all databases, including search terms, combinations and limits, are provided in the Supporting Information.

### 2.6 Selection Process

Following the completion of the literature search, duplicate records were identified and removed using EndNote Web (https://access.clarivate.com/login?app=endnote). All remaining records were then imported into the Rayyan web‐based platform (https://www.rayyan.ai) to facilitate additional duplicate removal and blinded screening of studies.

Study selection was conducted in two stages, in accordance with PRISMA guidelines [48]. In the first stage, three reviewers (J.T‐F., N.M‐R. and I.M.P.) independently screened titles and abstracts against the predefined eligibility criteria. In the second stage, the same reviewers independently assessed the full‐text reports of potentially eligible studies, applying the exclusion criteria. Full‐text articles were evaluated in their entirety.

**FIGURE 1:**
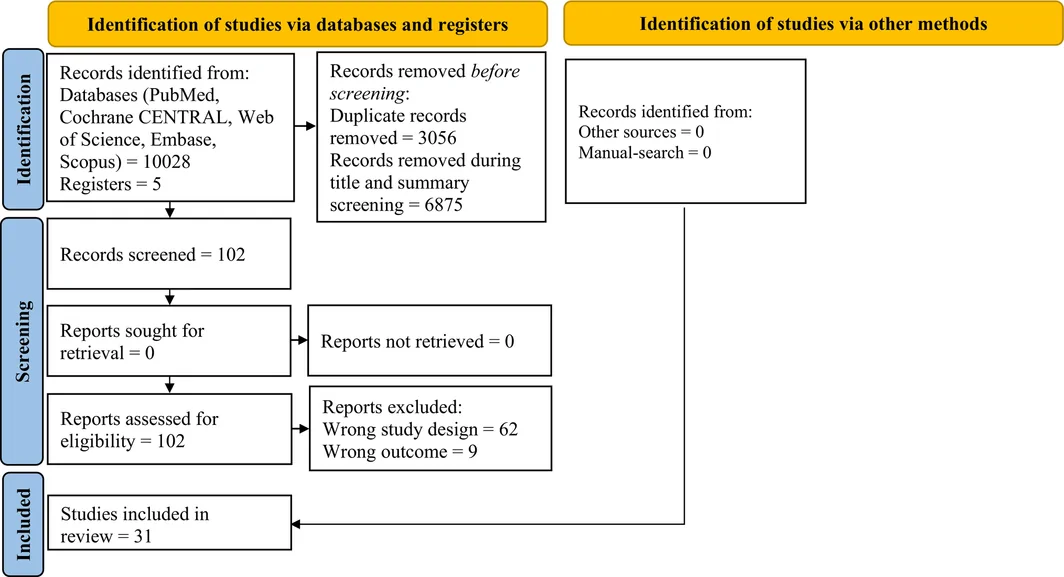
PRISMA flow chart of the literature screening. *Source:* Page et al. [48].

Agreement between reviewers at both stages was assessed using Cohen's κ statistic, with a prespecified threshold of κ ≥ 0.80 indicating substantial agreement [50]. Disagreements were initially addressed through discussion between reviewers and, when necessary, resolved by consultation with two senior reviewers (D.S.B. and R.F.G.). The final decision was always based on the full text reading. Reasons for exclusion at the full‐text stage are detailed in the PRISMA flow diagram (Figure 1).

### 2.7 Data Collection Process

Full texts of all potentially eligible studies were retrieved and assessed in detail. When necessary, corresponding authors were contacted to obtain additional information or clarify study details. Data extraction was performed by three reviewers (J.T‐F., N.M‐R. and I.M.P.) using a standardized data extraction form, in accordance with the recommendations of the Cochrane Handbook for Systematic Reviews of Interventions [51]. Reviewers were blinded to each other's assessments during the extraction process.

In cases where relevant data were unclear, incomplete or missing, attempts were made to contact the study authors by email over five consecutive weeks. If no response was obtained, numerical data were extracted from figures using WebPlotDigitizer (https://automeris.io/WebPlotDigitizer/), when applicable. Two senior reviewers (D.S.B. and R.F.G.) subsequently verified the accuracy and consistency of the extracted data.

For studies published in languages other than English, Google Translate (https://translate.google.com.br/) was used to assist with data extraction when necessary. Any discrepancies between reviewers were resolved through discussion and consensus.

Qualitative findings were synthesized and reported according to the predefined PICO framework. Details regarding the experimental design and methodological characteristics of each included study are presented in Table S1, whereas individual study outcomes and quality assessments are summarized in Table S2.

### 2.8 Risk of Bias Assessment

The risk of bias of in vivo animal studies was assessed using the SYRCLE Risk of Bias tool [52], in line with CAMARADES recommendations for the critical appraisal of experimental animal research [47]. This tool evaluates key domains related to internal validity, including sequence generation, baseline characteristics, allocation concealment, random housing, blinding of caregivers and outcome assessors, random outcome assessment, completeness of outcome data, selective outcome reporting and other potential sources of bias.

Each domain was independently judged by two reviewers as low risk, high risk or unclear risk of bias based on the information reported in the original studies. Disagreements were resolved through discussion and, when necessary, by consultation with two senior reviewers.

Consistent with current methodological guidance, no overall numerical score was calculated for in vivo studies. Instead, risk of bias was interpreted at the domain level and considered qualitatively in the synthesis and interpretation of the findings.

Risk of bias assessments were incorporated into the qualitative synthesis to support the interpretation of findings, but were not used as exclusion criteria. The results of the risk of bias evaluation are summarized in Table 3.

### 2.9 Incorporation of Risk of Bias Into Data Synthesis

The findings of included studies were synthesized with explicit consideration of their methodological quality and risk of bias. Results from studies judged to be at high risk of bias based on domain‐level assessments using the SYRCLE tool for in vivo animal studies [52], were interpreted with caution and were not given the same evidentiary weight as findings from studies with lower risk of bias.

Where inconsistencies in results were observed, differences in methodological rigour, experimental design or risk of bias domains were explored as potential explanatory factors. Risk of bias assessments were therefore used to contextualize heterogeneity across studies and to support a qualitative, rather than quantitative, integration of the evidence.

### 2.10 Synthesis Methods and Effect Measures

Results were synthesized qualitatively in accordance with the PRISMA 2020 statement [48], with no quantitative meta‐analysis performed. Given the substantial heterogeneity across preclinical studies in terms of experimental models, fluoride exposure characteristics, analytical methods and outcome definitions, a narrative synthesis was considered the most appropriate approach.

Descriptive findings were presented using structured text, figures and tables summarizing study selection, study characteristics, risk of bias within studies and results of individual studies. The qualitative synthesis was conducted following the Synthesis Without Meta‐analysis (SWiM) reporting guideline [53], with studies grouped and compared based on experimental design, model type, exposure parameters and outcome domains.

Risk of bias assessments were explicitly integrated into the synthesis and interpretation of findings. Results from studies judged to be at high risk of bias were interpreted with caution and were not given the same evidentiary weight as findings from studies with lower risk of bias. Consequently, conclusions were primarily informed by evidence derived from studies with low to moderate risk of bias, and no formal assessment of effect size or certainty of evidence through quantitative methods was undertaken.

### 2.11 Reporting Bias Assessment

Potential reporting biases were assessed qualitatively in accordance with the PRISMA 2020 statement and recommendations [48]. Given the absence of quantitative meta‐analysis and the heterogeneity of experimental models and outcomes, formal statistical assessments of reporting bias (e.g., funnel plot asymmetry or regression‐based tests) were not performed.

Instead, reporting bias was evaluated through a structured qualitative approach, considering the completeness and transparency of outcome reporting within individual studies, the consistency between stated objectives and reported results, and the presence of selectively reported outcomes. Particular attention was paid to discrepancies between methods and results sections, incomplete reporting of null or negative findings, and selective emphasis on statistically significant outcomes.

As part of the strategy to mitigate publication bias, grey literature sources were systematically searched alongside electronic bibliographic databases, as detailed in the search strategy section. In addition, the distribution of evidence across experimental models, exposure conditions, and outcome domains was examined to identify patterns suggestive of selective publication. The potential impact of reporting bias was considered in the interpretation of the findings and explicitly discussed as a limitation of the available preclinical evidence base.

## 3 Results

### 3.1 Study Selection

The searches identified 10 033 records. After removal of 3056 duplicates, 6977 records remained for title and abstract screening. Of these, 6875 records were excluded for not meeting the eligibility criteria. The remaining 102 articles underwent full‐text assessment, after which 71 studies were excluded for predefined reasons. Ultimately, 31 studies met the inclusion criteria and were included in the qualitative synthesis (Figure 1).

The individual quantitative results of the included studies are presented in Table S1. Overall, the studies demonstrated substantial heterogeneity in experimental design, animal models, exposure routes, target tissues and outcome measures. Despite this variability, several studies reported statistically significant alterations in the metallome across different organs following fluoride exposure, either alone or in combination with other elements. Due to the diversity of models and outcomes, results were synthesized qualitatively.

A qualitative synthesis of the direction and statistical significance of the effects observed across the included studies is presented in Table 2, in which the elements are grouped into essential metals and semi‐metals, toxic metals and metalloids, and other elements, and organized by tissue and anatomical compartment (mineralized tissues; soft tissues and organs; and circulating and excretory compartments), compiling the findings across all animal models. For each element and tissue, statistically significant increases or decreases (assessed against the corresponding fluoride‐free or metal‐only comparator reported in Table S1) are indicated by solid‐coloured arrows; where significant changes were reported in both directions across studies, the arrow shows the direction observed in the majority of studies. The predominant but non‐significant trend, where applicable, is represented by a dashed arrow, and the final column summarizes the net direction of the essential‐metal changes in each tissue. Overall, fluoride exposure was associated with tissue‐specific alterations in the concentration of essential and toxic elements, with both increases and decreases reported depending on the element, tissue, exposure dose and co‐exposure conditions. Considerable heterogeneity was observed across animal models and experimental designs; nevertheless, recurrent patterns emerged, particularly a predominant reduction of essential metals (most consistently Ca, Zn, Cu and Mn) across compartments, contrasted by an increased retention of the toxic metals Pb and Cd under co‐exposure. Due to this heterogeneity, findings were synthesized qualitatively rather than quantitatively.

**TABLE 2:** Direction of fluoride‐associated changes in essential, toxic and other elements, grouped by anatomical compartment. The final column gives the net direction of essential‐metal changes per tissue; highlighted cells mark tissues that deviate from the general depletion pattern.

Tissue/compartment	Essential metals								Toxic metals and metalloids				Other elements							Essential metals (net)
	Ca	Mg	K	Na	Fe	Zn	Cu	Mn	Pb	Cd	As	Al	Co	Se	Cr	Mo	Ni	Li	Sr	
***Mineralized tissues***																				
Bone	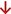	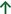	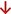		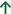	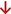	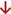	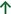	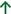	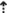				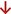			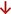			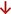
Tooth	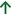	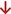				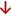	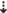	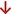	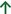											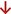
***Soft tissues/organs***																				
Liver					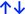	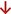	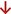	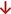	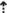	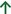	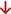		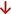							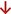
Kidney	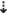	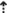			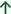	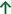	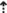	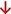	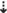	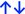	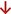		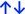				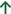			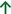
Renal cortex					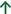	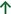	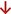	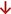												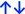
Heart	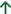	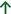	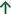	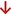	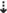	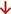	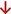	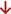					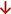							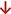
Brain					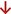	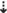	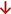	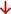				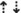								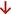
Lung						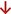	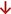	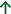			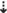		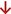							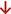
Spleen					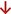	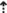	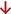	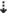												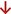
Pancreas					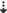	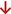	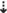	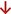												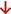
Muscle					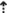	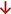	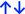	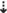					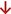				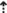			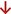
Colon										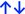										
Testis					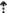	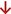	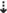	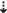												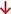
Aorta	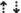	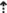																		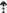
Gum	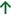	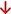				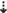	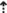	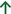												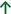
Wool					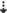	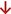	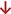	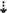		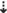			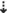				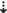			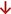
***Circulating & excretory***																				
Blood	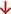	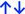			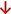	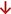	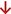	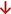	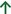	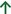			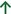							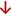
Plasma	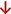	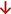			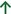	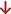	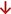	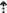						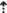						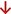
Serum	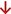	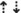			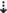	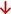	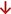	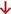					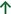		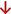	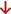		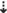	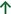	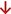
Urine	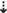	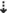				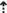	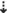			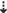	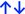	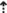								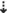
Faeces	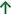	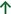			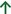	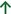	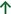													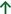
Bladder											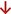									

*Note:* **Legend:** 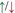 significant increase/decrease; 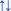 significant in both directions (arrow shows the direction reported by most studies); 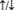 predominant non‐significant trend; blank, not assessed; colour indicates statistical significance. **Highlighted cells** in the last column mark the exceptions to the systemic essential‐metal depletion: fluoride accumulates essentials in the kidney and increases their faecal excretion (gum and renal cortex also deviate). Significance was confirmed against the fluoride‐free/metal‐only comparator (Table S1) and cross‐checked with Table S2 and Figure 2. Tooth combines enamel and dentin; Bone combines bone and femur; As includes inorganic and methylated species.

**FIGURE 2:**
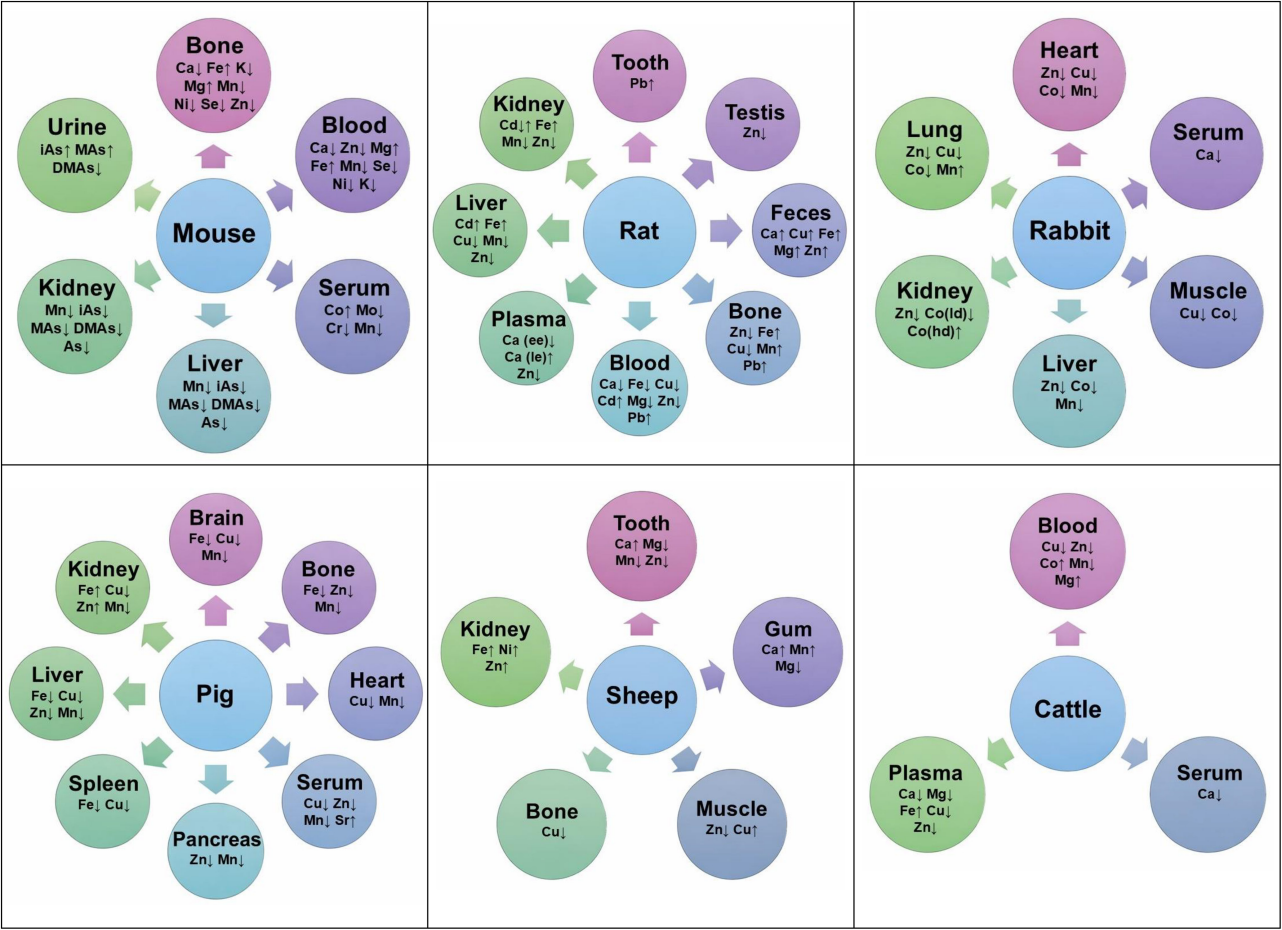
Qualitative evidence synthesis. Radial divergence plot depicting statistically significant variations in the metallome among different organs of animals exposed to fluoride. ee, early exposure; le, late exposure; ld, low dose; hd, high dose.

Figure 2 complements Table 2 by presenting these statistically significant variations in the metallome organized by animal model rather than by element, thereby preserving the species‐specific tissue patterns that Table 2 aggregates across species. For each species (mouse, rat, rabbit, sheep, pig and cattle), the figure depicts, for each tissue, the elements that varied significantly and the direction of variation.

### 3.2 Study Characteristics

This systematic review synthesizes four decades of preclinical evidence demonstrating that fluoride exposure is consistently associated with alterations in the metallome, as evidenced by changes in the concentration, distribution and tissue retention of metals and metalloids across various experimental models. The studies included in this review that met the inclusion criteria were published between 1981 and 2024, with 11 of them published in the last decade. This indicates significant progress and growing concern regarding the monitoring of essential and toxic elements and their impact on biological systems exposed to various environmental contaminants. The geographic distribution of these articles is as follows: India (*n* = 10), China (*n* = 9), Turkey (*n* = 4), Finland (*n* = 2), Brazil (*n* = 1), Denmark (*n* = 1), United Kingdom (*n* = 1), Poland (*n* = 1), United States (*n* = 1) and Mexico (*n* = 1).

As outlined in the inclusion criteria, only preclinical experimental studies in living animals (in vivo) are discussed in this review. The most frequently used animal model among the included studies was the rodent *Rattus norvegicus* , with Wistar (*n* = 9) and Sprague–Dawley (*n* = 6) being the predominant strains. Additionally, other species were employed in a smaller number of studies, including mice (albino or C57BL/6) (*n* = 7), rabbits (*n* = 2), pigs (*n* = 2), sheep (*n* = 3) and cattle (*n* = 2). Because the evidence base is dominated by rodents (22 of 31 studies), these patterns mainly reflect findings in rats and mice, with corroboration in large animals noted where available. Among essential elements, circulating Ca and tissue Zn and Cu were most often reduced, a pattern also observed in cattle and pigs, whereas Mn frequently rose in rodent bone, Mg showed inconsistent, context‐dependent changes (rising in bone in some rodent models but falling under calcium supplementation), and Fe was organ‐specific, tending to increase in rodent soft tissues while decreasing in blood and in several pig tissues. For toxic elements, all assessed in rodent co‐exposure studies, the direction depended on the element: Pb accumulation in calcified tissues increased under fluoride co‐exposure; arsenic tissue deposition decreased; blood Cd changed in a fluoride dose–dependent manner. These responses were strongly dependent on dose, duration of exposure, the tissue evaluated and the experimental context. Taken together, these findings indicate that the effects of fluoride on the metallome extend beyond dental and bone mineralization, providing the basis for the detailed analysis presented below.

The kidney and liver were the most frequently analysed soft tissues, assessed in 12 and 10 studies, respectively. Bone was the most commonly evaluated mineralized tissue (*n* = 12), with six of these studies specifically investigating the femur. Urine was analysed in six studies. Regarding blood samples, five studies evaluated plasma, six analysed serum, and six assessed whole blood. The heart was investigated in four studies. Other less frequently assessed tissues included the brain, muscle, lung, colon and testis (*n* = 2 each), as well as the aorta, faeces, pancreas, spleen, enamel, dentin, gingiva, wool and bladder (*n* = 1 each).

### 3.3 Risk of Bias Within Studies

In accordance with PRISMA 2020 recommendations, the risk of bias within individual studies was systematically evaluated using the SYRCLE tool, and the distribution of judgements across domains is summarized in Table 3. Overall, substantial variability in methodological reporting was observed across studies, with several domains frequently rated as having an unclear risk of bias due to insufficient reporting.

**TABLE 3:** SYRCLE's RoB tool domains.

Author, year	SYRCLE's RoB tool entries									
	1	2	3	4	5	6	7	8	9	10
Kanwar et al., 1981	Unc	Yes	Unc	Unc	Unc	Unc	Unc	Unc	Yes	Yes
Larsen et al., 1981	Unc	Yes	Unc	Unc	Unc	Unc	Unc	Unc	Yes	Yes
Singh and Kanwar, 1981	Unc	Yes	Unc	Unc	Unc	Unc	Unc	Unc	Yes	Yes
Singh, 1982	Unc	Yes	Unc	Unc	Unc	Unc	Unc	Unc	Yes	Yes
Ericsson et al., 1986	Unc	Yes	Unc	Unc	Unc	Unc	Unc	Unc	Yes	Yes
Cooke et al., 1990	Unc	Yes	Unc	Unc	Unc	Unc	Unc	Yes	Yes	Yes
Koskinen‐Kainulainen and Tuomisto, 1990	Unc	Yes	Unc	Unc	Unc	Unc	Unc	Yes	Yes	Yes
Krasowska and Wlostowski, 1992	Unc	Yes	Unc	Unc	Unc	Unc	Unc	Yes	Yes	Yes
Yu et al., 1992	Unc	Yes	Unc	Unc	Unc	Unc	Unc	Yes	Yes	Yes
Tao et al., 2005	Unc	Yes	Unc	Yes	Unc	Yes	Unc	Yes	Yes	No
Ranjan et al., 2008	Unc	Unc	Unc	Unc	Unc	Yes	Unc	Yes	Yes	Yes
Niu et al., 2009	Unc	Yes	Unc	Unc	Unc	Unc	Unc	Unc	Yes	Yes
Karademir, 2010	Unc	Yes	Unc	Unc	Unc	Unc	Unc	Unc	Yes	Yes
Sawan et al., 2010	Unc	Yes	Unc	Unc	Unc	Unc	Unc	Unc	Yes	Yes
Khandare et al., 2011	Unc	Yes	Unc	Unc	Unc	Unc	Unc	Yes	Yes	Yes
Ranjan et al., 2011	No	Yes	Unc	Unc	Unc	Unc	Unc	Yes	No	Yes
Chhabra et al., 2012	No	No	No	Unc	Unc	Unc	Unc	No	Yes	No
Chen et al., 2013	Yes	Yes	Unc	Unc	Unc	Unc	Unc	Yes	Yes	Yes
Zhang et al., 2013	Yes	Yes	Unc	Unc	Unc	Unc	Unc	Yes	Yes	Yes
Aydin et al., 2014	No	No	No	Unc	Unc	Unc	Unc	Yes	Yes	Yes
Khandare et al., 2015	Yes	Yes	Unc	Unc	Unc	Unc	Unc	Yes	Yes	Yes
Li et al., 2015	Yes	Yes	Unc	Unc	Unc	Unc	Unc	Yes	Yes	Yes
Çetin and Yur, 2016	Unc	No	No	No	Unc	No	Unc	Yes	Yes	No
Cetin et al., 2020	No	No	No	No	Unc	Unc	Unc	Yes	Yes	No
Dey Bhowmik et al., 2020	Yes	Yes	Unc	Unc	Unc	Unc	Unc	Yes	Yes	Yes
Li et al., 2023	Yes	Yes	Unc	Unc	Unc	Unc	Unc	Yes	Yes	Yes
Pena et al., 2023	Yes	Yes	Unc	Unc	Unc	Unc	Unc	Yes	Yes	Yes
Li et al., 2024a	Yes	Yes	Unc	Unc	Unc	Unc	Unc	Yes	Yes	Yes
Li et al., 2024b	Yes	Yes	Unc	Unc	Unc	Unc	Unc	Yes	Yes	Yes
Hu et al., 2024	Yes	Yes	Unc	Unc	Unc	Unc	Unc	Unc	No	Yes
Sawar et al., 2024	Yes	Yes	Unc	Unc	Unc	Unc	Unc	Unc	Yes	Yes

*Note:* Legend: Ten entries (domains) from SYRCLE's RoB tool: 1, Was the allocation sequence adequately generated and applied?; 2, Were the groups similar at baseline or were they adjusted for confounders in the analysis?; 3, Was the allocation to the different groups adequately concealed during?; 4, Were the animals randomly housed during the experiment?; 5, Were the caregivers and/or investigators blinded from knowledge which intervention each animal received during the experiment?; 6, Were animals selected at random for outcome assessment?; 7, Was the outcome assessor blinded?; 8, Were incomplete outcome data adequately addressed?; 9, Are reports of the study free of selective outcome reporting?; and 10, Was the study apparently free of other problems that could result in high risk of bias?

Abbreviation: Unc, unclear.

For Domain 1, 52% of the studies provided insufficient information to allow a clear judgement. In contrast, 35% of the studies were assessed as having a low risk of bias [44, 54, 55, 56, 57, 58, 59, 60, 61, 62, 63], whereas 13% were judged to have a high risk of bias [64, 65, 66, 67].

A predominance of low risk of bias was observed for Domains 2, 8, 9 and 10, with more than 80% of studies meeting the corresponding criteria. Specifically, Domain 2, which evaluates baseline group similarity and adjustment for confounding factors, showed a high risk of bias in four studies [65, 66, 67, 68], whereas one study lacked sufficient information for judgement [69].

For Domain 8 (incomplete outcome data), one study was classified as having a high risk of bias [65]. For Domain 9 (selective outcome reporting), two studies were assessed as having a high risk of bias [61, 64]. Regarding Domain 10, which addresses other potential sources of bias, four studies were judged not to be free from additional concerns that could introduce bias [65, 67, 68, 70].

In contrast, for Domains 3, 4 and 6, more than 80% of studies did not report sufficient methodological details, resulting in an ‘unclear risk of bias’ classification as defined by the SYRCLE tool.

Finally, Domains 5 and 7, which concern blinding of caregivers/investigators and blinding of outcome assessors, respectively, revealed substantial reporting limitations: all 31 included studies were rated as having an unclear risk of bias due to insufficient reporting.

## 4 Discussion

The central finding of this systematic review is that fluoride behaves as a systemic modifier of metallome organization rather than as an agent acting solely on mineralized tissues. Across four decades of preclinical studies, fluoride exposure was repeatedly associated with element‐specific changes of both essential and toxic elements among bone, soft tissues and circulating compartments, with the direction and magnitude of these changes depending on the element, the dose and duration of exposure, and the experimental context. These patterns, analysed element by element below, support the interpretation of fluoride as a systemic modifier of the metallome organization.

The data compiled in this review suggest that fluoride physicochemical behaviour in biological solutions has been overlooked so far. This fact opens a new window on how to view exposure to fluoride. Until now, fluoride exposure has been interpreted only from a toxicological perspective. From now on, it must be recognized that fluoride greatly alters the metallome of tissues, potentially interfering with a broad range of functions.

The biological consequences of fluoride exposure have traditionally been discussed in terms of oxidative stress, enzyme inhibition, mitochondrial dysfunction and tissue toxicity. The metallomic alterations observed across the included studies are also compatible with a more fundamental chemical phenomenon. Because fluoride is a highly electronegative ligand that participates in metal–ligand equilibria, its presence may alter the distribution of existing chemical species and thereby modify the metallome. In complex biological systems, metals continuously participate in dynamic equilibria involving mineral phases and biological molecules. To cite but a few examples in the case of proteins, optimal concentrations of essential metals are necessary for protein structure acquisition and maintenance [71, 72], cell membrane channel and transporter function [71, 73], optimal enzyme activities [74], mitochondrial inner membrane cytochrome function [75], control of redox balance [71] and many other metal‐dependent effects on proteins.

Viewed in this context, the recurrent changes in essential and toxic elements observed in experimental animals may reflect the biological expression of perturbations originating, at least in part, from shifts in underlying chemical equilibria. As stated in the introduction, fluoride can modify chemical equilibria involving metals, alter their speciation and influence the relative distribution of free and bound forms in aqueous systems [10, 11, 12]. It is important to note that changes in the metallome in several tissues after fluoride exposure are currently being investigated with a high degree of reliability. Nonetheless, subcellular changes in the metallome are still a great challenge and await technological [76] developments that will possibly allow us to envision the subtle changes in the many cellular functions that depend on metals.

### 4.1 Toxicokinetic and Exposure Context

In vertebrates, fluoride exposure occurs predominantly through ingestion, with absorption mainly in the small intestine and bioavailability influenced by dietary components, especially calcium; although a limited fraction is eliminated in faeces, approximately 45%–60% is excreted in urine, with a substantial portion retained in the organism, particularly in calcium‐rich tissues such as bones and teeth [15, 16, 77]. This overall profile of absorption, excretion and retention is consistent with the conclusions of the included studies, in which the kidney, liver, blood and mineralized tissues consistently emerge as relevant matrices for the assessment of metallomic alterations. Importantly, comparative toxicokinetic evidence indicates that rodents require fluoride concentrations in drinking water approximately 5× higher than those used in humans to achieve comparable plasma fluoride levels, reflecting interspecies differences in fluoride absorption, elimination and systemic metabolism [78]. This finding justifies the use of higher fluoride doses in rodent studies [46, 79, 80] when compared with studies conducted in large animal models, such as that of Ranjan et al., which compared cattle from endemic areas of fluoride contamination with healthy animals [69]. Accordingly, the toxicological relevance of these higher doses should be judged by their biological effects rather than by nominal fluoride concentrations alone. The occurrence of enamel fluorosis in teeth is an indicator that exposure to fluoride has exceeded the threshold, and the presence of fluoride disturbs normal enamel development. In this sense, fluorosis is not merely a dental finding but a visible marker of excessive systemic exposure, supporting the biological plausibility that the metallomic alterations observed in animal studies may also be relevant to human populations exhibiting the same phenotype.

Fluorotic enamel consistently exhibits increased retention of organic matrix [81]. Although previous studies report that fluoride does not directly alter enamel protease activity [82], a review by Bronckers et al. synthesized several in vivo and in vitro studies showing that fluoride can reduce ameloblast protease expression and activity and impair matrix degradation [83]. Interestingly, although in some in vitro tests using enamel proteases, fluoride did not show changes in protease activity, several metals like Zn, Pb and Cd decreased the activity of enamel proteases [84]. So, changes in metals caused by fluoride could also affect enamel proteases. This observation raises the possibility that fluoride may affect enamel protein removal either directly or indirectly through disturbances in the homeostasis of essential metals and the mineralizing environment, including calcium availability and pH regulation [85]. Zinc, for example, is required for the catalytic activity of MMP‐20, one of the major enzymes responsible for enamel matrix degradation during amelogenesis. In addition, KLK4, another protease essential for proper enamel maturation, has been shown to contain a specific zinc‐binding site through which micromolar Zn^2+^ modulates its enzymatic activity [84, 86]. Within this framework, fluoride‐induced alterations in zinc availability may impair the coordinated degradation and removal of enamel matrix proteins, thereby contributing to the increased organic content characteristic of fluorotic enamel.

### 4.2 Essential Metals

#### 4.2.1 Calcium

Across the included studies, fluoride was associated chiefly with a redistribution of calcium between mineralized and circulating compartments, with reductions in circulating Ca being the most consistent finding. In Swiss albino mice, prolonged exposure to NaF at 15 ppm for 4–8 months resulted in progressive reductions in bone Ca [57]. In Wistar rats, acute fluoride administration resulted in an early reduction in plasma Ca, with normalization or elevation at later evaluations, indicating a time‐dependent effect [87]. In Sprague–Dawley rats, Ericsson et al. showed that fluoride (30 ppm) combined with a Ca/Mg‐imbalanced diet resulted in lower plasma Ca levels compared with balanced diets, whereas Ca supplementation increased plasma Ca, highlighting the interaction between fluoride and the nutritional environment [88]. In large animals, reductions in serum Ca were described in cattle from endemic fluorosis areas compared with control animals [65, 69], and Ca depletion only in serum was reported in pigs by Khandare et al. [55]. Mechanistically, this pattern may reflect the high affinity of fluoride for calcium‐containing mineralized tissues, where fluoride is incorporated into apatite crystals through ionic exchange and promotes fluorapatite formation [89, 90]. Thus, fluoride exposure may disturb the dynamic exchange of Ca between mineralized tissues and the circulation, particularly under conditions of dietary Ca/Mg imbalance [88].

#### 4.2.2 Magnesium

Magnesium responses pointed to coordinated shifts between mineralized and soft tissues rather than a uniform direction of change. In Swiss albino mice under prolonged NaF exposure, bone Mg increased [57]. In Sprague–Dawley rats, Ca supplementation in the context of fluoride exposure reduced Mg, underscoring the dependence of Mg on the dietary and mineral environment [88]. In cattle from endemic fluorosis areas, alterations in Mg were also described compared with control animals [65, 69].

#### 4.2.3 Zinc, Copper and Manganese

These transition metals, grouped because they responded similarly across studies, showed predominantly reductions in soft and mineralized tissues, with manganese occasionally increasing in bone. In Wistar rats exposed to NaF at 25 ppm via drinking water, reductions in Zn and Cu in bone were observed concomitantly with an increase in Mn [91, 92]. In Swiss albino mice under prolonged NaF exposure, bone Zn was progressively reduced [57]. In Wistar rats chronically exposed to NaF at 100–200 ppm, consistent reductions in hepatic and renal Zn were described under prolonged exposure [79], whereas alterations in Cu and Mn were less uniform, varying according to the compartment and the experimental design. Under chronic exposure, consistent reductions in plasma Zn were also observed [79]. In rabbits exposed daily to fluoride, a decrease in essential metals (Zn, Cu, Co and Mn) in the liver and kidney was also recorded [64]. Alterations in reproductive tissues, such as the testis, were also reported after prolonged exposures, particularly for Zn, Fe and Cu, which showed declines, although these findings were less recurrent [79]. At the highest fluoride dose, serum Mn was markedly reduced (≈55%) in Swiss albino mice [93] (see ‘other elements’).

In cattle from endemic fluorosis areas, alterations in Zn and Cu were described [65, 69]. In pigs exposed to dietary fluoride, animals receiving the highest fluoride dose (150 ppm) exhibited marked reductions in Fe, Zn and Mn concentrations in bone tissue (approximately 45%, 22% and 41%, respectively), with no substantial change in Cu. In the liver, Fe, Cu, Zn and Mn decreased by about 40%, 52%, 22% and 54%, respectively. In contrast, the renal cortex showed increased Fe and Zn levels (approximately 45% and 36%), accompanied by reductions in Cu and Mn (around 24% and 49%), compared with control animals without added fluoride [70]. Also, in pigs, depletion of Fe, Cu, Zn and Mn was observed in serum and brain [70].

A plausible mechanism links several of these reductions to renal injury. The reduction of essential elements in bone may partly reflect a secondary consequence of fluoride‐induced renal injury. This interpretation is biologically plausible because the kidney is the main route of fluoride excretion and a recognized target organ of chronic fluoride toxicity, with experimental and human studies showing proximal renal tubular injury [94], oxidative stress, mitochondrial dysfunction [16, 17] and early renal dysfunction markers after fluoride exposure [22, 95]. This interpretation is also consistent with the findings of the present review, in which chronic fluoride exposure was associated with alterations in the kidney metallome. Renal dysfunction often results in reduced reabsorption of ions in the proximal renal tubule [96], a phenomenon that has already been described following fluoride exposure [94]. Therefore, increased urinary loss of trace elements may be expected and should be investigated in future studies. To maintain plasma trace element levels, these elements may be mobilized from bone, which could help explain the reduced bone levels of Zn, Cu and Mn [96]. Zn, in particular, may be stored in bone and mobilized under conditions of increased physiological demand [97].

Beyond this renal axis, fluoride may indirectly interfere with the homeostasis of these metals through oxidative stress and mitochondrial dysfunction. Previous studies indicate that fluoride exposure can reduce bone Zn and alter Cu distribution, decreasing its bone concentrations while increasing its renal levels, which supports the hypothesis of tissue‐specific redistribution rather than uniform systemic depletion [91, 98]. For Mn, this mechanism may be particularly relevant because fluoride has been associated with reduced expression of Mn‐SOD/SOD2, a manganese‐dependent mitochondrial enzyme, contributing to oxidative stress and mitochondrial dysfunction [99, 100]. Because Zn, Cu and Mn are essential elements that depend on tightly regulated homeostatic control, these mechanisms may contribute to their reduction or redistribution under fluoride exposure. Therefore, the alterations in Zn, Cu and Mn observed in the included studies probably reflect a combination of reduced renal reabsorption, changes between bone and soft tissues, and disruption of metal‐dependent antioxidant and mitochondrial pathways. In this context, an additional mechanistic hypothesis emerges from the integration of the metallomic findings summarized in this review with the extensive literature describing fluoride‐induced mitochondrial dysfunction. Mitochondrial energy metabolism depends on a large network of metalloproteins containing Fe, Cu, Mn, Zn and Mg, including cytochromes, iron–sulphur cluster proteins, antioxidant enzymes and ATP‐dependent systems. Because several of these elements were consistently altered across fluoride‐exposed animal models, it is conceivable that part of the mitochondrial dysfunction commonly attributed to fluoride [43] may arise secondarily from disturbances in metal availability and distribution. Under this framework, fluoride‐induced metallomic perturbation would not merely accompany oxidative stress and mitochondrial injury but could contribute mechanistically to their development. Although the studies included in this review were not designed to test this hypothesis directly, future investigations integrating metallomics with mitochondrial functional analyses may help determine whether alterations in essential metal homeostasis represent an upstream event in fluoride‐induced cellular toxicity.

#### 4.2.4 Iron

In contrast to the other essential transition metals, iron tended to increase in soft tissues and circulating compartments under prolonged exposure, with organ‐specific exceptions. In Swiss albino mice under prolonged NaF exposure, bone Fe increased [57]. In Wistar rats chronically exposed to NaF at 100–200 ppm, hepatic and renal Fe showed a progressive increase dependent on dose and time, indicating differential tissue redistribution of these essential elements [79]. Under chronic exposure, plasma Fe showed a moderate increase over time [79], revealing a pattern in which exposure duration intensifies the redistribution and tissue retention of specific elements. Testicular Fe declined after prolonged exposure, although less recurrently [79]. In cattle, alterations in Fe were described [65, 69], and in pigs, Fe was reduced in bone, liver, serum and brain but increased in the renal cortex, paralleling the pattern of the other transition metals (see zinc, copper and manganese) [70].

Mechanistically, this relative increase in Fe in soft tissues and circulating compartments may be related to fluoride‐induced oxidative stress and mitochondrial dysfunction, conditions that can disturb Fe handling and favour tissue retention over time [43, 101, 102].

Regarding the decrease in iron levels observed in specific tissues and organs, the literature has already shown that in rodents, at the cellular level, NaF can directly disrupt iron homeostasis by reducing the expression of ferritin heavy chain (Fth), the main iron‐storage protein, in maturation‐stage ameloblasts, thereby decreasing the cellular storage capacity and the fraction of iron retained in its stable, non‐reactive (oxide) form [103]. Therefore, it is important that future studies aiming to evaluate the effect of fluoride on Fe levels assess both the tissue and the cellular level, in order to generate robust information on the distribution of this element under exposure conditions.

### 4.3 Toxic Metals and Metalloids

#### 4.3.1 Lead

Lead displayed the clearest and most consistent pattern: fluoride co‐exposure increased Pb accumulation in calcified tissues, without evidence of compensatory redistribution. When dental tissues were evaluated, the effects became even more pronounced in contexts of co‐exposure to toxic metals. In Wistar rats exposed to Pb with or without fluoride, the presence of fluoride was associated with a marked increase in Pb concentrations in enamel, dentin and bone, indicating that mineralized tissues concentrate higher amounts of Pb (3× more Pb in the co‐exposure group when compared with the group exposed to Pb alone) [46]. Because both F and Pb accumulate in the calcified tissues, accumulating those elements up to 95% in adult animals and humans [104], it is possible that the fluoride–Pb interaction in bone may also have several other effects (besides the changes in Pb concentrations) not yet studied, for instance, changes in enzyme activity, and this is a very important area that deserves attention.

In co‐exposure contexts, blood is used as an internal dose biomarker, whereas bone is used as a total Pb burden biomarker [104]. Although both bone Pb and bone F concentrations are good biomarkers that might help compare outcomes across different studies using an internal dose biomarker, the same is not true for Pb in whole blood (for Pb) or for F in urine or plasma (for fluoride).

Differences in study design help explain divergent Pb findings. For instance, Pb in whole blood was very different in the study published by Sawan et al. [46] and Niu et al. [80] (both studies that co‐exposed rats to Pb and/or F). Both studies used Wistar rats exposed via drinking water. However, the rats were exposed from the beginning of gestation onward [46] or after delivery [80], and the exposure duration (until 60 days in Niu et al. and 81 days in Sawan et al.), fluoride source (sodium fluoride vs. fluorosilicic acid) and administered doses (150 ppm F and 300 ppm Pb in Niu et al., and 100 ppm F and 30 ppm Pb in Sawan et al.) differed. Suggesting the fluoride source may play a role in the uptake of Pb in the blood. Interestingly, although the study by Niu et al. did not show increased amounts of Pb in the co‐exposure group (as Sawan et al. did show), these authors showed a worsening of the neurological parameters tested in the group of rats co‐exposed to F + Pb, and according to the description of the authors in their study: ‘learning abilities and hippocampus glutamate levels were significantly decreased by F and Pb individually and the combined interaction of F and Pb’. A recent animal study also involving co‐exposure to F and Pb, but using lower fluoride doses than those previously cited (50 ppm F and 30 ppm Pb), demonstrated increased lead concentration in the brain tissue, as well as in the kidneys and bone, of co‐exposed animals compared with controls [105].

#### 4.3.2 Cadmium and Arsenic

For cadmium, the direction of the effect on blood concentrations was governed by the fluoride dose. In Sprague–Dawley rat models co‐exposed to Cd via drinking water, alterations in blood Cd levels were observed in a fluoride dose–dependent manner. At lower fluoride concentrations, an increase in blood Cd was reported in animals exposed to 20 ppm of fluoride [54], whereas at higher fluoride concentrations, a decrease in blood Cd levels was observed in animals exposed to 100 ppm of fluoride [44]. These studies, however, did not evaluate mineralized tissues.

Arsenic was the only element for which chemical speciation was reported, and fluoride co‐exposure was associated with lower tissue deposition. In mice, in addition to changes in essential metals, the effect of fluoride on As metabolism was highlighted; co‐exposure to fluoride and As was associated with reductions in total concentrations and in different arsenic species in the liver and kidney, compared with exposure to As alone, indicating lower tissue deposition of the metalloid in the presence of fluoride [59].

Although the mechanisms remain unclear, these findings suggest that fluoride may alter the toxicokinetics of Cd and As rather than producing a uniform increase or decrease in accumulation. This interpretation is consistent with studies that observed that fluoride exposure reduced Cd levels in the kidney and liver [58] and decreased As concentrations in these same organs [59]. Therefore, dose‐dependent changes in renal handling, tissue retention or excretion may help explain why Cd increased in blood at lower fluoride exposure but decreased at higher exposure, whereas As showed reduced hepatic and renal deposition in the presence of fluoride.

### 4.4 Other metals (Al, Co, Mo, Cr)

A smaller group of less frequently investigated elements was reported in single studies, broadening the spectrum of fluoride effects. In Wistar rats co‐exposed to fluoride and Al, higher brain Al concentrations were observed in the group exposed to fluoride alone compared with the co‐exposed group, although the latter still showed higher levels than controls [56]. Karademir [93] showed that, in Swiss albino mice, serum alterations induced by fluoride exposure were observed not only in classical elements such as Mn but also in less commonly investigated trace elements, including Co, Mo and Cr, with the highest fluoride dose (40 mg/L) associated with decreases in Mn (≈55%), Mo (≈66%) and Cr (≈13%), alongside an increase in Co of approximately 21%, relative to control animals. A decrease in Co was also recorded in the liver and kidney of rabbits exposed daily to fluoride [64].

### 4.5 Compartment‐Dependent Changes and the Differential Handling of Essential Versus Toxic Elements

Fluoride exposure promotes alterations in the metallome, with greater robustness in mineralized tissues and more heterogeneous responses in soft tissues and circulatory matrices. In small animal models, particularly rats and mice, mineralized tissues emerge as the compartments most sensitive to fluoride‐induced alterations, especially with respect to the essential minerals Ca, P and Mg, which constitute the largest inorganic fraction of these matrices.

Overall, the reviewed evidence indicates that fluoride exposure is associated with element‐specific and compartment‐dependent redistribution rather than uniform changes across tissues. Increased retention of Ca in mineralized tissues, particularly bone, has been reported in studies incorporating dietary modulation [106], whereas reductions in circulating Ca following fluoride exposure have been consistently observed in plasma or serum, supporting a change between skeletal and circulating compartments [87]. Similarly, alterations in skeletal Mg, accompanied by changes in plasma and excretory pathways, indicate coordinated shifts between mineralized and soft tissues [88]. For Mn, several experimental models showed increased concentrations in bone concomitant with reductions in serum, liver or kidney at higher fluoride doses, reinforcing a partial complementary redistribution across compartments [92]. However, this pattern was not consistent across all elements or exposure conditions, as Zn, Cu and Fe exhibited heterogeneous and organ‐specific responses depending on dose and target tissue [70, 98]. In contrast, for toxic metals such as Pb, studies assessing mineralized tissues demonstrated marked Pb accumulation in bone, enamel and dentin under fluoride co‐exposure, without evidence of compensatory redistribution [46, 107].

Findings in large animals further support this interpretation. The cattle findings, in particular, point to an integrated dysregulation of the essential metallome under chronic environmental exposure to fluoride [65, 69]. Taken together, findings in large animals are consistent with the patterns observed in small animal models, particularly regarding the sensitivity of mineralized tissues and the redistribution of essential metals in the liver and in the kidney, indicating the translational relevance of the metallomic effects of fluoride observed in rodents for chronic exposures in larger species.

A unifying interpretation of these apparently opposite directions is that the net effect of fluoride on a given element depends less on its valence than on whether the element is under active homeostatic control. The essential cations (both the macrominerals Ca and Mg and the trace transition metals Fe, Zn, Cu and Mn) are maintained by tightly regulated absorption, storage and excretion, so that a disturbance of mineral handling tends to be corrected through loss. Under fluoride exposure, two excretory routes appear to act in the same direction: Reduced proximal‐tubular reabsorption favours urinary loss [94, 96], whereas the formation of poorly soluble fluoride–cation complexes in the intestinal lumen reduces absorption and increases faecal elimination of divalent cations [108]. To preserve circulating concentrations, these elements may additionally be mobilized from skeletal stores [96, 97]. This combination is consistent with the pattern emphasized throughout this review, in which essential elements decline in bone while transiently rising in excretory organs, as illustrated by the increase in renal‐cortex Fe and Zn in pigs even as their bone and liver concentrations fell [70]. By contrast, the toxic, non‐essential cations Pb and Cd lack an equivalent regulated excretory pathway; once absorbed, they are retained rather than eliminated. Because Pb^2+^ closely mimics Ca^2+^, it substitutes for calcium within the apatite lattice, and the fluoride‐driven conversion of hydroxyapatite into fluorapatite, a denser, less soluble and resorption‐resistant mineral [90], would be expected to stabilize this incorporation, turning bone into a more efficient and less reversible sink for Pb under co‐exposure. In this framework, fluoride does not act in opposite ways on essential and toxic elements; rather, the same disturbance of renal and mineral handling produces net depletion of the elements the organism can regulate and net retention of those it cannot.

At the membrane‐transport level, the divalent metal transporter DMT1 offers a shared route of cellular uptake for several of these cations, with an in vitro selectivity that favours Cd^2+^ over Fe^2+^ and Mn^2+^ [109, 110]. Because DMT1 expression rises when iron availability falls, a fluoride‐induced perturbation of iron homeostasis could secondarily enhance cadmium uptake, providing a plausible mechanism for the increase in blood Cd observed at lower fluoride doses [111]. Lead, in contrast, appears to enter cells largely through DMT1‐independent pathways [112], in keeping with its handling being dominated by incorporation into mineralized tissue rather than by shared divalent‐cation transport. The opposite, dose‐dependent behaviour of Cd at higher fluoride concentrations indicates that additional processes, such as altered renal retention or competition at absorptive sites, also contribute and remain to be clarified.

The biological response to fluoride also appears to be dose‐ and context‐dependent. Thus, the data reported in these studies indicate that dose, exposure time, route of exposure and dietary context decisively influence the magnitude and direction of metallomic alterations. Although hormesis was not directly assessed in the studies included in this review, complementary experimental evidence suggests that the biological response to fluoride may follow a dose‐ and time‐dependent adaptive pattern [113]. In this context, prolonged exposure to low doses, particularly those closer to concentrations used in artificially fluoridated drinking water, has been associated with adaptive responses linked to improved metabolic parameters and redox balance [114]. By contrast, higher doses, more consistent with exposure scenarios in endemic fluorosis areas, tend to be associated with metabolic disruption and oxidative imbalance [113]. Genetic background also appears to modulate these responses, contributing to differential susceptibility across experimental models [115]. In co‐exposure settings involving fluoride and other metals, the possibility of hormetic responses remains insufficiently explored and should be investigated more rigorously, particularly through the use of internationally certified reference materials and low‐background blanks [105].

The brain deserves particular attention as a target for future metallomic studies. In addition to Ca, which is essential and found in high concentrations in the brain, various parts of the brain require different concentrations of trace elements such as Fe, Zn, Cu and Mg (among others) for proper functioning [116]. An important direction for future studies on fluoride exposure is the investigation of the brain metallome with attention to regional differences among brain structures. As trace elements are being studied with more accurate techniques and with lower blanks, it is becoming clear that the role of certain elements in the brain is very important.

### 4.6 Methodological and Analytical Considerations

Besides those facts, the divergent results in the metallome probably stem from the fact that most studies are not comparable, due to differences in exposure regimens, fluoride doses, animal age and other environmental low‐dose factors, such as endocrine disruptors and nanoplastics, for example, that may disrupt ion transport, cell metabolism and fate [117, 118, 119, 120].

Additionally, although it is mandatory for laboratories that routinely measure clinical samples for toxic metals to participate in interlaboratory proficiency programmes to obtain laboratory accreditation, this is not necessarily the case for laboratories that measure trace elements like Pb and/or F in animal samples, which may, at least in part, account for the differences in results across studies. Moreover, the lack of use of certified material from the same matrix as the tissues used for metal determination in the chemical analyses, and also the analytical chemistry parameters for metal determinations, such as the limit of detection (LOD) and limit of quantification (LOQ), were not described in most of the studies included in this review.

Previous studies describe the interaction of fluoride with mineralized tissues, particularly through its incorporation into calcium‐rich apatite structures [15], as well as its association with Pb accumulation in calcified tissues under co‐exposure conditions [46, 105]. Although the studies included in this review were not designed to directly evaluate these mechanisms, the multielement patterns observed for Ca, Mg, Zn, Cu, Mn, Fe, Pb, Cd, As and Al in tissues and fluids are compatible with an element‐specific influence of fluoride on elemental distribution and retention. In addition, previously described associations between fluoride exposure, alterations in metal‐dependent antioxidant systems and oxidative stress [121, 122] reinforce the biological relevance of the observed changes in Zn, Mn, Cd and Pb concentrations, particularly in contexts of chronic exposure and co‐exposure.

### 4.7 Translational Relevance and Risk Assessment

From a translational perspective, current preclinical evidence should not be interpreted as definitive proof of toxic synergy but rather as an indication that fluoride exposure may act as a modifier of metal homeostasis under specific experimental conditions.

The relevance of these experimental observations is underscored by the existence of large human populations chronically exposed to fluoride in areas of endemic fluorosis, regions that also account for most of the studies synthesized in this review. China and India are two of the countries from which stem most studies on the study of F exposure and its health consequences, particularly due to the high prevalence of endemic fluorosis in their populations. In India, the first reports of the disease date back to the 1930s, with a notable study conducted in the Nellore district, where an association was observed between the consumption of F‐rich groundwater and changes in the bones and teeth of the local population [123]. Since then, fluorosis has become a persistent public health problem in the country, affecting millions of people and being linked to neurological impacts [124].

In China, concerns about fluorosis intensified following the identification of outbreaks in regions where the population used coal contaminated with F, especially in the Guizhou province [125]. Additionally, the chronic and excessive consumption of F‐rich teas, such as brick tea, commonly consumed in Tibetan communities and rural areas, represents an additional source of exposure in endemic regions [125, 126]. In light of this, the Chinese government has structured, since the 1960s, specific programmes for controlling, monitoring and mitigating fluorosis [126], which have been reinforced by epidemiological studies that have highlighted the widespread distribution and clinical severity of the issue [127].

The persistence of endemic fluorosis in these countries motivates not only public health surveillance but also a robust scientific production aimed at understanding the toxic mechanisms of F and its interactions with essential minerals and metals [128].

The high proportion of unclear risk judgements does not necessarily imply flawed study conduct; rather, it reflects incomplete methodological transparency. Nevertheless, such limitations may compromise the interpretability and reproducibility of the findings and should be considered when concluding the evidence base. Future in vivo studies would benefit from improved adherence to reporting guidelines such as ARRIVE [129], alongside transparent reporting of randomization and blinding procedures, to enhance methodological rigour and reduce the risk of bias.

### 4.8 Future Perspectives and Gaps

Despite decades of experimental research on fluoride toxicity and extensive evidence regarding the adverse effects of metals and semi‐metals, the present systematic review highlights substantial and persistent knowledge gaps concerning their interactions in preclinical models. The available evidence remains fragmented, methodologically heterogeneous and largely hypothesis‐generating rather than confirmatory.

One of the most critical gaps identified is the lack of standardized experimental designs. Studies varied widely in fluoride dose, exposure duration, route of administration, animal strain, age at exposure and target tissues, severely limiting cross‐study comparability. In many cases, fluoride concentrations exceeded environmentally or clinically relevant exposure scenarios, reducing translational relevance and complicating risk interpretation.

Analysis of the review findings suggests that studies with larger sample sizes tend to present lower variability, whereas studies with small sample sizes, as observed in several reports included in the review, tend to show greater variability, which may increase the risk of type II error. Therefore, even studies conducted in different animal species that did not detect significant differences should, in future investigations, include larger sample sizes to improve the reliability of the findings.

Another major limitation concerns the analytical scope of the available evidence. Most included studies relied on total elemental concentrations and did not directly assess metal speciation, oxidation states, subcellular localization or binding to biological ligands such as proteins and metabolites. Therefore, although the observed changes in metal and semi‐metal concentrations support the hypothesis that fluoride can modify metal/metalloid homeostasis, they do not allow direct conclusions regarding metal–biomolecule interactions or specific metalloprotein/metallometabolite alterations. Future studies should integrate advanced analytical approaches capable of distinguishing chemical species, intracellular localization and metal–biomolecule complexes to clarify the mechanistic basis of fluoride–metal interactions.

A further gap concerns the near‐absence of tissue‐resolved metallomic data for dental hard tissues. Although several of the elements discussed above are decisive for the cells that form enamel and dentin, no study has yet profiled the metallome of these tissues in fluoride‐exposed animals. The existing evidence is element‐by‐element and mechanistic rather than compositional. Fluoride disturbs Ca^2+^ signalling and mitochondrial function in enamel‐forming cells [102] echoing the mechanisms invoked above for soft tissues, and magnesium is actively removed during enamel maturation and inhibits crystal growth in opposition to fluoride [130] a behaviour distinct from the skeletal Mg increase reported in bone in this review. For toxic metals, fluoride co‐exposure increases lead concentrations in enamel and dentin [46] the same pattern seen in bone in this review. Fluoride exposure also lowers enamel mineral content while raising its organic matrix, an effect that has been tentatively linked to interference with zinc‐dependent matrix proteolysis [131]. Even comprehensive reviews of fluoride's effects on amelogenesis focus on mineralization, pH regulation and matrix proteolysis rather than on elemental composition [83]. When composition has been examined experimentally, analyses have targeted the bulk organic and inorganic fractions of fluorotic enamel [81, 132] or a limited set of elements, for example, iron storage in ameloblasts [103] and increased lead retention in the superficial enamel and dentin of rats co‐exposed to fluoride and lead [46], rather than encompassing a multielement profile. In humans, nanoscale chemical characterization has shown that, in severe dental fluorosis, subtle but measurable differences in elemental composition can be detected, including increased Fe and F content in the mineral nanocrystals [133]. Even so, most studies analysing the elemental composition of human teeth have focused on general biomonitoring rather than on fluorotic teeth from individuals with well‐characterized fluoride exposure, including exposure parameters such as plasma fluoride levels. A comprehensive multielement (metallome‐level) profile of mineralized dental tissues under characterized fluoride exposure is therefore still lacking. Future studies should apply integrative metallomic approaches to enamel and dentin in controlled fluoride‐exposure models to determine whether the redistribution patterns observed in soft tissues and bone extend to dental hard tissues.

Mechanistic understanding also remains limited. Although oxidative stress, mitochondrial dysfunction and interference with metal‐dependent enzymes have been proposed as shared pathways, few studies explicitly investigated mechanistic endpoints linking fluoride exposure to altered metal homeostasis. Integrative experimental designs incorporating redox biology, metal transporters and signalling pathways are needed to move beyond descriptive associations.

Importantly, nutritional status has been largely neglected as a modifying factor. Interactions between fluoride and essential elements such as calcium, magnesium, iron, zinc and selenium were inconsistently explored, despite strong biological plausibility. Controlled studies incorporating dietary manipulation are essential to clarify whether observed changes reflect toxic displacement, adaptive redistribution or secondary effects of mineral imbalance.

Finally, the review underscores the absence of dose–response frameworks and life‐stage–specific analyses. Few studies evaluated developmental windows of susceptibility, reversibility of effects or long‐term consequences of early‐life exposure. Addressing these gaps is critical for improving the translational relevance of preclinical findings and for informing future human biomonitoring and risk assessment strategies.

Collectively, future research should prioritize harmonized experimental protocols, environmentally relevant exposure scenarios, mechanistic endpoints and integrative analytical approaches. Such advances are necessary to establish causal relationships and to gain a deeper understanding of the biological significance of fluoride–metal interactions.

## 5 Conclusion

This systematic review consolidates preclinical evidence indicating that fluoride exposure is associated with alterations in the concentrations of metals and semi‐metals across multiple experimental models and biological matrices. Although the direction and magnitude of these changes vary substantially among studies, the findings collectively suggest that fluoride can influence elemental distribution and accumulation under controlled laboratory conditions.

Nevertheless, the available evidence is characterized by pronounced heterogeneity and variable methodological quality, limiting the ability to draw definitive or generalizable conclusions. As such, current observations should be interpreted with caution and viewed as indicative of potential interactions rather than conclusive demonstrations of toxic synergy or risk. This review provides a structured and critical synthesis of existing data, offering a clearer framework for interpreting preclinical findings on fluoride–metal interactions.

## Author Contributions

Conceptualization/design: D.S.B. and R.F.G.; Methodology: N.M‐R., J.T‐F., D.S.B. and R.F.G.; Validation: F.B.J., D.S.B. and R.F.G.; Data collection: N.M‐R., J.T‐F. and I.M.P.; Data analysis/interpretation: N.M‐R., J.T‐F., D.S.B., I.M.P., E.B.G., M.A.R.B., F.B.J. and R.F.G.; Writing – original draft preparation: N.M‐R., J.T‐F., D.S.B., I.M.P., E.B.G., M.A.R.B., F.B.J. and R.F.G.; Writing – critical revision and editing: N.M‐R., J.T‐F., D.S.B. and R.F.G.; Funding acquisition: R.F.G.; Supervision: R.F.G. All authors have read and agreed to the published version of the manuscript.

## Funding

This work was supported by the Coordenação de Aperfeiçoamento de Pessoal de Nível Superior ‐ Brasil (CAPES), the National Council for Scientific and Technological Development (CNPq) and the São Paulo Research Foundation (FAPESP; 2022/00626‐6, 2022/00629‐5, 2025/15660‐3, 2025/12654‐2).

## Conflicts of Interest

The authors declare no conflicts of interest.

## Supporting information


**Table S1:** Experimental design characteristics and individual quantitative outcomes of the included studies.
**Table S2:** Qualitative synthesis of direction and consistency of effects across included studies.

## Data Availability

Data sharing not applicable to this article as no datasets were generated or analysed during the current study.
