# Supplementary material for: Fluoride as a Modifier of Metallome Homeostasis: A Systematic Review of Animal Studies
Source: Basic Clin Pharmacol Toxicol. 2026 Sep 4;139(4):e70295. doi: 10.1111/bcpt.70295 (PMC13543053; doi:10.1111/bcpt.70295)
Supplement: Supplementary file 1 [file BCPT-139-0-s001.docx]

***Basic & Clinical Pharmacology & Toxicology***

**Fluoride as a Modifier of Metallome Homeostasis: A Systematic Review of Animal Studies**

***Fluoride and Metallome Homeostasis***

Natalia Macedo-Ribeiro^a#^, Jonas Tostes-Figueiredo^a#^, Davi da Silva Barbirato^b#^, Isabel Maria Porto^c^, Eliude Barbosa Gomes^a^, Erico Marlon Moraes Flores^d^ Marília Afonso Rabelo Buzalaf^e^, Fernando Barbosa Junior^f^, Raquel Fernanda Gerlach^a^*

^a^Department of Basic and Oral Biology, Faculty of Dentistry of Ribeirao Preto, University of Sao Paulo (FORP/USP), Av. Cafe, S/N- Ribeirao Preto, 14040-904, Sao Paulo, Brazil.

^b^Department of Oral Surgery and Periodontology, Faculty of Dentistry of Ribeirao Preto, University of Sao Paulo (FORP/USP), Av. Cafe, S/N- Ribeirao Preto, 14040-904, Sao Paulo, Brazil.

^c^Department of Morphology and Children’s Clinic. School of Dentistry at Araraquara. Sao Paulo State University- UNESP (FOAr/UNESP). Rua Humaitá, 1680, 14801-903, Araraquara, Sao Paulo, Brazil.

^d^Chemistry Department, Federal University of Santa Maria, 97105-900, SantaMaria, RS, Brazil (UFSM)

^e^Department of Biological Sciences, Bauru School of Dentistry, University of São Paulo, Bauru, São Paulo, Brazil (FOB/USP)

^f^Laboratory of Toxicology, School of Pharmaceutical Sciences of Ribeirao Preto, University of Sao Paulo (FCFRP/USP), Ribeirao Preto, SP, Brazil.

#These authors contributed equally.

***Correspondence**

Raquel Fernanda Gerlach. Department of Basic and Oral Biology, Faculty of Dentistry of Ribeirao Preto, University of Sao Paulo (FORP/USP), Avenida do Cafe Av., Ribeirao Preto, 14040-904, Sao Paulo, Brazil. Phone number: +55 (16) 33154136 | Fax: +55 (16) 33154788. Email address: rfgerlach@forp.usp.br (Gerlach, R.F.).

| **TABLE S1**. Experimental design characteristics and individual quantitative outcomes of the included studies. | | | | | | | | |
| --- | --- | --- | --- | --- | --- | --- | --- | --- |
| **Author, year** | **Pre-clinical model** | | **Target tissue/organ** | | **Individual results \| statistically significant differences (*/#)** | | | |
|  |  |  |  |  | **Intervention** | | **Comparator** | |
| **WISTAR RATS** | | | | | | | | |
| Kanwar et al., 1981 | Water intake | | Liver from Wistar rats | | Zn, Cu, and Mn μg/g (ppm)  **Group I- F 10 mg/L (ppm):**  Zn (8; 190 ± 28.5), Cu (8; 23.4 ± 3.83), and Mn (8; 22.2 ± 3.43)  **Group II- F 25 mg/L (ppm):**  Zn (8; 144 ± 15.3)*, Cu (8; 17.8 ± 3)*, and Mn (8; 17.1 ± 3.31)*  (N; mean ± SD) | | Zn, Cu, and Mn μg/g (ppm)  **Water without F:**  Zn (8; 203 ± 21.7), Cu (8; 22.4 ± 2.17), and Mn (8; 22.6 ± 2.23)  (N; mean ± SD) | |
|  |  |  | Kidney from Wistar rats | | Zn, Cu, and Mn μg/g (ppm)  **Group I- F 10 mg/L (ppm):**  Zn (8; 177 ± 22.9), Cu (8; 22.8 ± 2.8), and Mn (8; 21.2 ± 2.87)  **Group II- F 25 mg/L (ppm):**  Zn (8; 226 ± 21.2)*, Cu (8; 22.3 ± 3.1), and Mn (8; 14.1± 1.8)*  (N; mean ± SD) | | Zn, Cu, and Mn μg/g (ppm)  **Water without F:**  Zn (8; 168 ± 17.8), Cu (8; 20.2 ± 2.3), and Mn (8; 18.9 ± 2.61)  (N; mean ± SD) | |
|  |  |  | Femur from Wistar rats | | Zn, Cu, and Mn μg/g (ppm)  **Group I- F 10 mg/L (ppm):**  Zn (8; 374 ± 49.7), Cu (8; 9.2 ± 1.83), and Mn (8; 11.9 ± 2.1)  **Group II (F 25 mg/L (ppm):**  Zn (8; 309 ± 30.6)*, Cu (8; 5.17 ± 1.12)*, and Mn (8; 15 ± 2.6)*  (N; mean ± SD) | | Zn, Cu, and Mn μg/g (ppm)  **Water without F:**  Zn (8; 355 ± 29.7), Cu (8; 8.8 ± 1.31), and Mn (8; 10.9 ± 1.37)  (N; mean ± SD) | |
| Larsen et al., 1981 | Intraperitoneal injection | | Plasma from Wistar rats | | 6-9 days of life Ca (mmol/L)  **Group I (intraperitoneal injection of NaF 10 mg/Kg):**  5 min. (2.65 ± 0.14)*, 15 min. (2.49 ± 0.25)*, 60 min. (2.66 ± 0.19)*, 4 h. (2.73 ± 0.37)*, and 4 days (2.74 ± 0.13)*  **Group II (intraperitoneal injection of NaF 20 mg/Kg):**  5 min. (2.42 ± 0.23)*, 15 min. (2.30 ± 0.24)*, 60 min. (2.22 ± 0.29)*, 4 h. (2.31 ± 0.38)*, and 4 days (2.73 ± 0.05)*  **Group III (intraperitoneal injection of NaF 40 mg/Kg):**  5 min. (2.68 ± 0.09)*, 15 min. (2.53 ± 0.13)*, 60 min. (1.87 ± 0.20)*, 4 h. (2.48 ± 0.02)*, and 4 days: (2.89 ± 0.22)*  90-95 days of life Ca (mmol/L)  **Group IV (intraperitoneal injection of NaF 10 mg/Kg):**  5 min. (2.37 ± 0.16)*, 15 min. (2.40 ± 0.13)*, 60 min. (2.39 ± 0.06)*, 4 h. ( 2.53 ± 0.12)*, and 4 days (2.51 ± 0.04)*  **Group V (intraperitoneal injection of NaF 20 mg/Kg):**  5 min. (2.30 ± 0.15)*, 15 min. (2.26 ± 0.06)*, 60 min. (2.08 ± 0.06)*, 4 h. (2.29 ± 0.12)*, and 4 days (2.51 ± 0.04)*  **Group VI (intraperitoneal injection of NaF 40 mg/Kg):**  5 min. (2.36 ± 0.03)*, 15 min. (2.21 ± 0.12)*, 60 min. (1.57 ± 0.22)*, 4 h. (1.02 ± 0.13)*, and 4 days (2.47 ± 0.07)*  (mean ± SD) | | 6-9 days of life Ca (mmol/L)  Intraperitoneal injection of physiological saline  **Group VII (Intraperitoneal injection of saline):** (1; 2.86 ± 0.22)  **Group VIII (Intraperitoneal injection of saline):** (1; 2.57 ± 0.25)  **Group IX (Intraperitoneal injection of saline):** (1; 2.99 ± 0.17)  90-95 days of life Ca (mmol/L)  Intraperitoneal injection of physiological saline  **Group X (Intraperitoneal injection of saline):** (1; 2.46 ± 0.12)  **Group XI (Intraperitoneal injection of saline):** (1; 2.49 ± 0.05)  **Group XII (Intraperitoneal injection of saline):** (1, 2.44 ± 0.03)  (N; mean ± SD) | |
| Singh and Kanwar, 1981 | Water intake | | Kidney from Wistar rats | | Zn, Cu, and Mn μg/g (ppm)  **Group I (F 10 ppm):**  Cu (8; 22 ± 3), Mn (8; 21 ± 3), and Zn (8; 180 ± 23)  **Group II (F 25 ppm):**  Cu (8; 22 ± 3), Mn (8; 14 ± 2)*, and Zn (8; 230 ± 21)*  (N; mean ± SD) | | Zn, Cu, and Mn μg/g (ppm)  **Water without F:**  Cu (8; 20 ± 2), Mn (8; 19 ± 3), and Zn (8; 170 ± 18)  (N; mean ± SD) | |
|  |  |  | Femur from Wistar rats | | Zn, Cu, and Mn μg/g (ppm)  **Group I (F 10 ppm):**  Cu (8; 9.2 ± 1.8), Mn (8; 12 ± 2), and Zn (8; 370 ± 50)  **Group II (F 25 ppm):**  Cu (8; 5.2 ± 1.1)*, Mn (8; 15 ± 3)*, and Zn (8; 310 ± 31)*  (N; mean ± SD) | | Zn, Cu, and Mn μg/g (ppm)  **Water without F:**  Cu (8; 8.8 ± 1.3), Mn (8; 11 ± 1), and Zn (8; 360 ± 30)  (N; mean ± SD) | |
| Koskinen-Kainulainen and Tuomisto, 1990 | Water gavage | | Serum from Wistar rats | | Ca, and Mg (μg/mL)  **Group I (F 30 mg/Kg):**  Ca (6; 119 ± 4.3), and Mg (6; 42.1 ± 4.3)  **Group II (F 30 mg/Kg + Mg 30 mg/Kg):**  Ca (6; 114 ± 3), and Mg (6; 46.4 ± 2.2)  (N; Mean ± SD) | | Ca, and Mg (μg/mL)  **Group III (water without F and Mg):**  Ca (6; 109 ± 2.6), and Mg (6; 44.9 ± 1.3)  **Group IV (water without F + Mg 30 mg/Kg):**  Ca (6; 123 ± 4.5), and Mg (6; 46.5 ± 2.9)  (N; Mean ± SD) | |
|  |  |  | Bone from Wistar rats | | Ca, and Mg (μg/mg)  **Group I (F 30 mg/Kg):**  Ca (6; 231 ± 11.8), and Mg (6; 4.89 ± 2.05)  **Group II (F 30 mg/Kg + Mg 30 mg/Kg):**  Ca (6; 236 ± 7.4), and Mg (6; 5.17 ± 0.95)  (N; Mean ± SD) | | Ca, and Mg (μg/mL)  **Group III (water without F and Mg):**  Ca (6; 223 ± 5.8), and Mg (6; 4.91 ± 1.08)  **Group IV (water without F + Mg 30 mg /Kg):**  Ca (6; 228 ± 8.3), and Mg (6; 4.8 ± 1.73)  (N; Mean ± SD) | |
|  |  |  | Urine from Wistar rats | | Ca, and Mg (μg/24h)  **Group I (F 30 mg/Kg):**  Ca (6; 951 ± 145), and Mg (6; 1406 ± 218)  **Group II (F 30 mg/Kg + Mg 30 mg/Kg):**  Ca (6; 797 ± 102), and Mg (6; 3252 ± 274)  (N; Mean ± SD) | | Ca, and Mg (μg/24h)  **Group III (water without F and Mg):**  Ca (6; 500 ± 122), and Mg (6; 2276 ± 218)  **Group IV (water without F + Mg 30 mg /Kg):**  Ca (6; 834 ± 150), and Mg (6; 3826 ± 207)  (N; Mean ± SD) | |
|  |  |  | Feces from Wistar rats | | Ca, and Mg (μg/24h)  **Group I (F 30 mg/kg):**  Ca (6; 4462 ± 1283), and Mg (6; 576 ± 0.169)  **Group II (F 30 mg/Kg + Mg 30 mg/Kg):**  Ca (6; 3043 ± 1108), and Mg (6; 537 ± 180)  (N; Mean ± SD) | | Ca, and Mg (μg/24h)  **Group III (water without F and Mg):**  Ca (6; 3386 ± 899), and Mg (6; 410 ± 111)  **Group IV (water without F + Mg 30 mg /Kg):**  Ca (6; 2504 ± 714), and Mg (6; 468 ± 130)  (N; Mean ± SD) | |
|  |  |  | Heart from Wistar rats | | K, Na, Ca, and Mg (mg/g)  **Group I (F 30 mg/kg):**  K (6; 16.4 ± 1.58)^#^, Na (6; 3.05 ± 0.39)^#^*, Ca (6; 0.38 ± 0.15)*, and Mg (6, 1.17 ± 0.08)  **Group II (F 30 mg/kg + Mg 30 mg/kg):**  K (6, 15.5 ± 1.96), Na (6, 3.21 ± 0.70)*, Ca (6; 0.22 ± 0.13)^#^*, and Mg (6; 1.29 ± 0.23)*  (N; Mean ± SD) | | K, Na, Ca, and Mg (mg/g)  **Group III (Water without F and Mg):**  K (6; 15 ± 2.29), Na (6; 4.14 ± 0.50), Ca (6; 0.06 ± 0.01), and Mg (6; 1 ± 0.07)  **Group IV (Water + Mg 30 mg /Kg):**  K (6; 13.8 ± 1.39), Na (6; 3.81 ± 0.68), Ca (6; 0.05 ± 0.01), and Mg (6; 1.05 ± 0.43)  (N; Mean ± SD) | |
| Krasowska and Wlostowski, 1992 | Water intake | | Plasma from Wistar rats | | Fe and Zn (μg/mL)  **Group I (NaF 100 ppm):**  Fe 6 weeks (7; 2.36 ± 0.48), Fe 16 weeks (7; 2.17 ± 0.13), Zn 6 weeks (7; 0.97 ± 0.05)*, and Zn 16 weeks (7; 0.75 ± 0.04)*,  **Group II (NaF 200 ppm):**  Fe 6 weeks (7; 2.54 ± 0.28), Fe 16 weeks (7; 2.28 ± 0.31), Zn 6 weeks: (7; 1.03 ± 0.04)*, and Zn 16 weeks (7; 0.73 ± 0.07)*,  (N; mean ± SE) | | Fe and Zn (μg/mL)  **Group III (water without F):**  Fe 6 weeks (7; 2.48 ± 0.26), Fe 16 weeks (7; 2.15 ± 0.19), Zn 6 weeks (7; 1.34 ± 0.12), and Zn 16 weeks (7; 1.20 ± 0.05),  (N; mean ± SE) | |
|  |  |  | Liver from Wistar rats | | Fe, Zn (μg/mL), Cu, and Mn (μg/g [ppm])  **Group I (NaF 100 ppm):**  Fe 6 weeks (7; 202 ± 20.1), Fe 16 weeks (7; 329 ± 28.4), Zn 6 weeks (7; 66.7 ± 4), Zn 16 weeks (7; 46.5 ± 4)*, Cu 6 weeks (7; 23.9 ± 0.6), Cu 16 weeks (7; 19.7 ± 3), Mn 6 weeks (7; 7.3 ± 0.4), and Mn 16 weeks (7; 7 ± 0.5)  **Group II (NaF 200 ppm):**  Fe 6 weeks (7; 236 ± 30.6), Fe 16 weeks (7; 421 ± 31.3)*, Zn 6 weeks (7; 61.4 ± 5), Zn 16 weeks (7; 37.4 ± 4)*, Cu 6 weeks (7; 21 ± 1.3), Cu 16 weeks (7; 18.4 ± 2.3), Mn 6 weeks (7; 6.8 ± 0.2), and Mn 16 weeks (7; 6.6 ± 0.6)  (N; mean ± SE) | | Fe, Zn (μg/mL), Cu, and Mn (μg/g [ppm])  **Group III (water without F):**  Fe 6 weeks (7; 207 ± 29.1), Fe 16 weeks (7; 316 ± 26.2), Zn 6 weeks (7; 64 ± 5), Zn 16 weeks (7; 67.1 ± 8), Cu 6 weeks (7; 19.1 ± 2.5), Cu 16 weeks (7; 20.9 ± 4.9), Mn 6 weeks (7; 7.2 ± 0.4), and Mn 16 weeks (7; 6.9 ± 0.2)  (N; mean ± SE) | |
|  |  |  | Kidney from Wistar rats | | Fe, Zn (μg/mL), Cu, and Mn (μg/g [ppm])  **Group I (NaF 100 ppm):**  Fe 6 weeks (7; 158 ± 14), Fe 16 weeks (7; 275 ± 20.7)*, Zn 6 weeks (7; 61.4 ± 4), Zn 16 weeks (7; 37.9 ± 3)*, Cu 6 weeks (7; 46.9 ± 2.3), Cu 16 weeks (7; 54.8 ± 2.9), Mn 6 weeks (7; 1.7 ± 0.2), and Mn 16 weeks (7; 1.8 ± 0.3)  **Group II (NaF 200 ppm):**  Fe 6 weeks (7; 237 ± 15.3)*, Fe 16 weeks (7; 328 ± 15)*, Zn 6 weeks (7; 42.2 ± 4)*, Zn 16 weeks (7; 41.2 ± 3)*, Cu 6 weeks (7; 48.9 ± 3.8), Cu 16 weeks (7; 57.9 ± 3.4), Mn 6 weeks (7; 1.8 ± 0.2), and Mn 16 weeks (7; 1.5 ± 0.1)  (N; mean ± SE) | | Fe, Zn (μg/mL), Cu, and Mn (μg/g [ppm])  **Group III (water without F):**  Fe 6 weeks (7; 187 ± 12.3), Fe 16 weeks (7; 248 ± 10.5), Zn 6 weeks (7; 60.7 ± 4), Zn 16 weeks (7; 59.5 ± 5), Cu 6 weeks (7; 54.9 ± 5.3), Cu 16 weeks (7; 60.8 ± 4.1), Mn 6 weeks (7; 1.8 ± 0.3), and Mn 16 weeks (7; 1.8 ± 0.4)  (N; mean ± SE) | |
|  |  |  | Femur from Wistar rats | | Fe, and Zn (μg/mL)  **Group I (NaF 100 ppm):**  Fe 6 weeks (7; 72.9 ± 6)*, Fe 16 weeks (7; 67.7 ± 5), Zn 6 weeks (7; 198 ± 17), Zn 16 weeks (7; 173 ± 16),  **Group II (NaF 200 ppm):**  Fe 6 weeks (7; 81.5 ± 10)*, and Fe 16 weeks (7; 96.6 ± 7.8)*, Zn 6 weeks (7; 211 ± 19)*, Zn 16 weeks (7; 235 ± 17)*,  (N; mean ± SE) | | Fe, and Zn (μg/mL)  **Group III (water without F):**  Fe 6 weeks (7; 66.2 ± 6), and Fe 16 weeks (7; 62.8 ± 2.7), Zn 6 weeks (7; 187 ± 8), Zn 16 weeks (7; 151 ± 15),  (N; mean ± SE) | |
|  |  |  | Testis from Wistar rats | | Fe, Zn (μg/mL), Cu, and Mn (μg/g [ppm])  **Group I (NaF 100 ppm):**  Fe 6 weeks (7; 90.2 ± 10.9), Fe 16 weeks: (7; 155 ± 15.8)*, Zn 6 weeks (7; 169 ± 8), Zn 16 weeks (7; 134 ± 6)*, Cu 6 weeks (7; 17.3 ± 1.7), Cu 16 weeks (7; 16.6 ± 1.8), Mn 6 weeks (7; 5.2 ± 1.0), and Mn 16 weeks (7; 5.4 ± 0.6)  **Group II (NaF 200 ppm):**  Fe 6 weeks (7; 93.9 ± 12.4), Fe 16 weeks (7; 173 ± 20)*, Zn 6 weeks (7; 139 ± 7)*, Zn 16 weeks (7; 120 ± 8)*, Cu 6 weeks (7; 17.2 ± 1.9), Cu 16 weeks (7; 18.1 ± 2.1), Mn 6 weeks (7; 4.7 ± 0.7), and Mn 16 weeks (7; 5 ± 1)  (N; mean ± SE) | | Fe, Zn (μg/mL), Cu, and Mn (μg/g [ppm])  **Group III (water without F):**  Fe 6 weeks (7; 80.7 ± 7.7), Fe 16 weeks (7; 142 ± 14.4), Zn 6 weeks (7; 181 ± 7), Zn 16 weeks (7; 170 ± 6), Cu 6 weeks (7; 21.9 ± 2), Cu 16 weeks (7; 20.2 ± 3.9), Mn 6 weeks (7; 5.2 ± 0.9), and Mn 16 weeks (7; 5.6 ± 0.3)  (N; mean ± SE) | |
| Niu et al., 2009 | Water intake | | Blood from Wistar rats | | Pb (μg/L)  **Group I (NaF 150 mg/L [ppm])**: 8; 47.5 ± 3.31*  **Group II (NaF 150 mg/L [ppm] + Lead acetate 300 mg/L):** 8; 132 ± 6.39*  (N; mean ± SE) | | Pb (μg/L)  **Group III (water without F and Pb):** 8; 42.5 ± 2.32  **Group IV (water with lead acetate 300 mg/L):** 8; 170 ± 4.42  (N; mean ± SE) | |
| Sawan et al., 2010 | Water intake | | Whole blood from Wistar rats | | Pb μg/g (ppm)  **Group I (F 100 mg/L [ppm]):** 10; < 0.5  **Group II (F 100 mg/L [ppm] + Pb 30 mg/L [ppm]):** 10; 76.7 ± 11^#^  (N; mean ± SD) | | Pb (μg/dL)  **Group III (water without F and Pb):** 10; < 0.5  **Group IV (Pb 30 mg/L [ppm]):** 10; 22.6 ± 8.5  (N; mean ± SD) | |
|  |  |  | Enamel surface from Wistar rats | | Pb μg/g (ppm)  **Group I (F 100 mg/L [ppm]):** 10; 19.8  **Group II (F 100 mg/L [ppm] + Pb 30 mg/L [ppm]):** 10; 4369 ± 1353^#^  (N; mean ± SD) | | Pb μg/g (ppm)  **Group III (water without F and Pb):** 10; < 0.5  **Group IV (Pb 30 mg/L [ppm]):** 10; 1768 ± 1892  (N; mean ± SD) | |
|  |  |  | Whole dentine from Wistar rats | | Pb μg/g (ppm)  **Group I (F 100 mg/L [ppm]):** 10; 0.31 ± 0.32  **Group II (F 100 mg/L [ppm] + Pb 30 mg/L [ppm]):** 10; 8.5 ± 2.0^#^  (N; mean ± SD) | | Pb μg/g (ppm)  **Group III (water without F and Pb):** 10; < 0.5  **Group IV (Pb 30 mg/L [ppm]):** 10; 4.9 ± 1.7  (N; mean ± SD) | |
|  |  |  | Bone surface from Wistar rats | | Pb μg/g (ppm)  **Group I (F 100 mg/L [ppm]):** 10; 0.49 ± 0.68  **Group II (F 100 mg/L [ppm] + Pb 30 mg/L [ppm]):** 10; 28 ± 10.6^#^  (N; mean ± SD) | | Pb μg/g (ppm)  **Group III (water without F and Pb):** 10; < 0.5  **Group IV (Pb 30 mg/L [ppm]):** 10; 9 ± 3.7  (N; mean ± SD) | |
|  |  |  | Whole bone from Wistar rats | | Pb μg/g (ppm)  **Group I (F 100 mg/L [ppm]):** 10; < 0.5  **Group II (F 100 mg/L [ppm] + Pb 30 mg/L [ppm]):** 10; 14.2 ± 2.6^#^  (N; mean ± SD) | | Pb μg/g (ppm)  **Group III (water without F and Pb):** 10; < 0.5  **Group IV (Pb 30 mg/L [ppm]):** 10; 6.8 ± 1.7  (N; mean ± SD) | |
| Li et al., 2015 | Water intake | | Urine from Wistar rats | | Al (mg/L)  **Group I (F 50 mg/L [ppm]):** Al (10; 2.01 ± 0.03)  **Group II (F 50 mg/mL [ppm] + Al 100 mg/L [ppm]):** Al (10; 4.8 ± 0.20)*  (N; mean ± SD) | | Al (mg/L)  **Group III (water without F):** Al (10; 1.43 ± 0.08)  **Group IV (water without F + Al 100 mg/L [ppm]):** Al (10; 4.33 ± 0.25)  (N; mean ± SD) | |
|  |  |  | Brain from Wistar rats | | Al (mg/L)  **Group I (F 50 mg/L [ppm]):** Al (10; 0.39 ± 0.03)  **Group II (F 50 mg/mL [ppm] + Al 100 mg/L [ppm]):** Al (10; 0.54 ± 0.04)*  (N; mean ± SD) | | Al (mg/L)  **Group III (water without F):** Al (10; 0.21 ± 0.02)  **Group IV (water without F + Al 100 mg/L [ppm]):** Al (10; 0.91 ± 0.06)  (N; mean ± SD) | |
| Sawar et al., 2024 | Water intake | | Blood from Wistar rats | | Ca, Fe, Zn, Mg, and Cu μg/g (ppm)  **Group I (F 50 ppm):**  Ca (6; 14.8 ± 1.07)*, Fe (6; 12.9 ± 1.9)*, Zn (6; 0.73 ± 0.05)*, Mg (6; 37.5 ± 1.46)*, and Cu (6; 2.43 ± 0.17)*  **Group II (F 100 ppm):**  Ca (6; 12.5 ± 1.46)*, Fe (6; 6.21 ± 0.12)*, Zn (6; 0.66 ± 0.04)*, Mg (6; 35.6 ± 2.2)*, and Cu (6; 2.36 ± 0.06)*  (N; mean ± SEM) | | Ca, Fe, Zn, Mg, and Cu μg/g (ppm)  **Water without F:**  Ca (6; 16.5 ± 1.85), Fe (6; 15.7 ± 3.04), Zn (6; 2.59 ± 0.39), Mg (6; 63.5 ± 9.88), and Cu (6; 3 ± 0.36)  (N; mean ± SEM) | |
|  |  |  | Stool from Wistar rats | | Ca, Fe, Zn, Mg, and Cu μg/g (ppm)  **Group I (F 50 ppm):**  Ca (6; 11 ± 0.54)*, Fe (6; 11.3 ± 0.44), Zn (6; 7.86 ± 0.33)*, Mg (6; 20.7 ± 1.12)*, and Cu (6; 10.2 ± 0.24)*  **Group II (F 100 ppm):**  Ca (6; 13.2 ±1.8)*, Fe (6; 12.6 ± 0.37)*, Zn (6; 9.13 ± 0.22)*, Mg (6; 22.6 ± 2.86)*, and Cu (6; 11. ± 0.31)*  (N; mean ± SEM) | | Ca, Fe, Zn, Mg, and Cu μg/g (ppm)  **Water without F:**  Ca (6; 10.4 ± 0.46), Fe (6; 10.1 ± 0.91), Zn (6; 7.26 ± 2.67), Mg (6; 16.8 ± 1.99), and Cu (6; 9.2 ± 0.81)  (N; mean ± SEM) | |
| **SPRAGUE-DAWLEY RATS** | | | | | | | | |
| Ericsson et al., 1986 | Diet  Balance (Ca 0.43%, P 0.43%, Mg 485 ppm, K 0.48%) or Imbalance (Ca 0.12%, P 0.82%, Mg 98 ppm, K 0.19%) with water intake | | Plasma from Sprague-Dawley rats | | Ca, and Mg μg/mL (ppm)  **Group I (Diet imbalance + F 30 ppm):**  Ca (7-11; 49.6 ± 3.6), and Mg (7-11; 14.2 ± 2.4)  **Group II (Diet imbalance + 0.2 % Ca + F 30 ppm):**  Ca (7-11; 90 ± 12), and Mg (15; 6.3 ± 2.1)  **Group III (Diet imbalance + Mg 200 ppm + F 30 ppm):**  Ca (7-11; 54.4 ± 10.4), and Mg (7-11; 23.5 ± 3.2)  (N; mean ± SD) | | Ca, and Mg μg/mL (ppm)  **Group IV (Diet balance + water without F):**  Ca (7-11; 85.2 ± 14.4), and Mg (7-11; 18.2 ± 2.1)  **Group V (Diet imbalance + water without F):**  Ca (7-11; 54.4 ± 11.6), and Mg (7-11; 14.7 ± 1.9)  **Group VI (Diet imbalance + water without F + Ca 0.2 %):**  Ca (7-11; 82.4 ± 13.2), and Mg (7-11; 6 ± 2.6)  **Group VII (Diet imbalance + water without F + Mg 200 ppm):**  Ca (7-11; 59.6 ± 6.8), and Mg (7-11; 21.9 ± 2.7)  (N; mean ± SD) | |
|  |  |  | Aorta from Sprague-Dawley rats | | Ca, and Mg μg/g (ppm)  **Group I (Diet imbalance + F 30 ppm):**  Ca (14-16; 352 ± 67), and Mg (15; 218 ± 29)  **Group II (Diet imbalance + 0.2 % Ca + F 30 ppm):**  Ca (14-16; 452 ± 58), and Mg (14-16; 241 ± 19)  **Group III (Diet imbalance + Mg 200 ppm + F 30 ppm):**  Ca (14-16; 343 ± 36), and Mg (14-16; 248 ± 26)  (N; mean ± SD) | | Ca, and Mg μg/g (ppm)  **Group IV (Diet balance + water without F):**  Ca (14-16; 371 ± 123), and Mg (14-16; 266 ± 39)  **Group V (Diet imbalance + water without F):**  Ca (14-16; 297 ± 35), and Mg (14-16; 235 ± 15)  **Group VI (Diet imbalance + water without F + Ca 0.2 %):**  Ca (14-16; 452 ± 55), and Mg (14-16; 227 ± 22)  **Group VII (Diet imbalance + water without F + Mg 200 ppm):**  Ca (14-16; 430 ± 106), and Mg (14-16; 240 ± 22)  (N; mean ± SD) | |
|  |  |  | Heart from Sprague-Dawley rats | | Ca, and Mg μg/g (ppm)  **Group I (Diet imbalance + F 30 ppm):**  Ca: (14-16; 65 ± 10), and Mg (14-16; 1017 ± 35)  **Group II (Diet imbalance + 0.2 % Ca + F 30 ppm):**  Ca (14-16; 96 ± 17), and Mg (14-16; 1013 ± 57)  **Group III (Diet imbalance + Mg 200 ppm + F 30 ppm):**  Ca (14-16; 64 ± 7), and Mg (14-16; 1085 ± 50)  (N; mean ± SD) | | Ca, and Mg μg/g (ppm)  **Group IV (Diet balance + water without F):**  Ca (14-16; 102 ± 21), and Mg (14-16; 1059 ± 41)  **Group V (Diet imbalance + water without F):**  Ca (14-16; 85 ± 13), and Mg (14-16; 1012 ± 38)  **Group VI (Diet imbalance + water without F + Ca 0.2 %):**  Ca (14-16; 113 ± 20), and Mg (14-16; 1018 ± 49)  **Group VII (Diet imbalance + water without F + Mg 200 ppm):**  Ca (14-16; 67 ± 7), and Mg (14-16; 1042 ± 50)  (N; mean ± SD) | |
|  |  |  | Kidney from Sprague-Dawley rats | | Ca, and Mg μg/g (ppm)  **Group I (Diet imbalance + F 30 ppm):**  Ca (14-16; 1010 ± 888), and Mg (14-16; 893 ± 33)  **Group II (Diet imbalance + 0.2 % Ca + F 30 ppm):**  Ca (14-16; 7404 ± 7630), and Mg (15; 869 ± 71)  **Group III (Diet imbalance + Mg 200 ppm + F 30 ppm):**  Ca (14-16; 513 ± 210), and Mg (14-16; 920 ± 65)  (N; Mean ± SD) | | Ca, and Mg μg/g (ppm)  **Group IV (Diet balance + water without F):**  Ca (14-16; 371 ± 44), and Mg (14-16; 858 ± 43)  **Group V (Diet imbalance + water without F):**  Ca (14-16; 6039 ± 8744), and Mg (14-16; 921 ± 160)  **Group VI (Diet imbalance + water without F + Ca 0.2 %):**  Ca (14-16; 3669 ± 2946), and Mg:(14-16; 832 ± 65)  **Group VII (Diet imbalance + water without F + Mg 200 ppm):**  Ca (14-16; 623 ± 418), and Mg (14-16; 905 ± 42)  (N; Mean ± SD) | |
|  |  |  | Femur from Sprague-Dawley rats | | Ca, and Mg μg/g (ppm)  **Group I (Diet imbalance + F 30 ppm):**  Ca (13-16; 364 ± 8), and Mg (13-16; 0.89 ± 0.07)  **Group II (Diet imbalance + 0.2 % Ca + F 30 ppm):**  Ca (13-16; 355 ± 4), and Mg (13-16; 0.26 ± 0.03)  **Group III (Diet imbalance + Mg 200 ppm + F 30 ppm):**  Ca (13-16; 348 ± 4), and Mg (13-16; 1.15 ± 0.11)  (N; Mean ± SD) | | Ca, and Mg μg/g (ppm)  **Group IV (Diet balance + water without F):**  Ca (13-16; 363 ± 11), and Mg (13-16; 0.84 ± 0.04)  **Group V (Diet imbalance + water without F):**  Ca (13-16; 359 ± 17), and Mg (13-16; 0.71 ± 0.09)  **Group VI (Diet imbalance + water without F + Ca 0.2 %):**  Ca (13-16; 361 ± 8), and Mg (13-16; 0.25 ± 0.03)  **Group VII (Diet imbalance + water without F + Mg 200 ppm):**  Ca (13-16; 362 ± 19), and Mg (13-16; 0.93 ± 0.04)  (N; Mean ± SD) | |
| Yu et al., 1992 | Diet | | Plasma from Sprague-Dawley rats | | Se (μM)  **Group I (F 2 mg/Kg + Se 0.05 mg/Kg):** 5; 5.18 ± 0.27  **Group II (F 2 mg/Kg + Se 3 mg/Kg):** 5; 7.58 ± 0.89  **Group III (F 2 mg/Kg + Se 5 mg/Kg):** 5; 8.17 ± 1.05  **Group IV (F 75 mg/Kg + Se 0.05 mg/Kg):** 5; 5.1 ± 0.21  **Group V (F 75 mg/Kg + Se 3 mg/Kg):** 5; 7.88 ± 0.45  **Group VI (F 75 mg/Kg + Se 5 mg/Kg):** 5; 8.01 ± 0.75  **Group VII (F 150 mg/Kg + Se 0.05 mg/Kg):** 5; 4.98 ± 0.49  **Group VIII (F 150 mg/Kg + Se 3 mg/Kg):** 5; 7.28 ± 0.39  **Group IX (F 150 mg/Kg + Se 5 mg/Kg):** 5; 8.12 ± 0.57  (N; mean ± SD) | | Se (μM)  **Diet without F and Se:** (5; < 0.001)  (N; below detection level) | |
|  |  |  | Liver from Sprague-Dawley rats | | Se (nmol/g)  **Group I (F 2 mg/Kg + Se 0.05 mg/Kg):** 5; 29.5 ± 3.1  **Group II (F 2 mg/Kg + Se 3 mg/Kg):** 5; 91.9 ± 13.9  **Group III (F 2 mg/kg + Se 5 mg/kg):** 5; 186 ± 53.1  **Group IV (F 75 mg/Kg + Se 0.05 mg/Kg):** 5; 29.1 ± 6  **Group V (F 75 mg/Kg + Se 3 mg/Kg):** 5; 84.2 ± 12.7  **Group VI (F 75 mg/Kg + Se 5 mg/Kg):** 5; 133 ± 37.5  **Group VII (F 150 mg/Kg + Se 0.05 mg/Kg):** 5; 27.5 ± 2.4  **Group VIII (F 150 mg/Kg + Se 3 mg/Kg):** 5; 73.8 ± 6.4  **Group IX (F 150 mg/Kg + Se 5 mg/Kg):** 5; 123 ± 32.6  (N; mean ± SD) | | Se (nmol/g)  **Diet without F and Se:** (5; < 0.001)  (N; below detection level) | |
|  |  |  | Kidney from Sprague-Dawley rats | | Se (nmol/g)  **Group I (F 2 mg/Kg [ppm] + Se 0.05 mg/Kg [ppm]):** 5; 35.6 ± 5.9  **Group II (F 2 mg/Kg [ppm] + Se 3 mg/Kg [ppm]):** 5; 157 ± 26.1  **Group III (F 2 mg/Kg [ppm] + Se 5 mg/Kg [ppm]):** 5; 565 ± 393  **Group IV (F 75 mg/Kg [ppm] + Se 0.05 mg/Kg [ppm]):** 5; 38.7 ± 4.5  **Group V (F 75 mg/Kg [ppm] + Se 3 mg/Kg [ppm]):** 5; 127 ± 23.1  **Group VI (F 75 mg/Kg [ppm] + Se 5 mg/Kg [ppm]):** 5; 366 ± 78.7  **Group VII (F 150 mg/Kg [ppm] + Se 0.05 mg/Kg [ppm]):** 5; 41.3 ± 4.8  **Group VIII (F 150 mg/Kg [ppm] + Se 3 mg/Kg [ppm]):** 5; 160 ± 30.2  **Group IX (F 150 mg/Kg [ppm] + Se 5 mg/Kg [ppm]):** 5; 863 ± 443  (N; mean ± SD) | | Se (nmol/g)  **Diet without F and Se:** (5; < 0.001)  (N; below detection level) | |
| Chen et al., 2013 | Water intake | | Blood from Sprague–Dawley rats | | Cd (μg/L)  **Group I (F 20 mg/L):** 6; 0.12 ± 0.07  **Group II (Cd 50 mg/L+ F 20 mg/L):** 6; 5.5 ± 1.5*^#^  (N; mean ± SD) | | Cd (μg/L)  **Group III (water without F and Cd):** 6; 0.13 ± 0.11  **Group IV (water without F + Cd 50 mg/L):** 6; 3.18 ± 0.7  (N; mean ± SD) | |
|  |  |  | Urinary from Sprague–Dawley rats | | Cd (μg/mmol creatinine)  **Group I (F 20 mg/L):** 6; 0.22 ± 0.18  **Group II (Cd 50 mg/L+ F 20 mg/L):** 6; 4.64 ± 1.92*  (N; mean ± SD) | | Cd (μg/mmol creatinine)  **Group III (water without F and Cd):** 6; 0.26 ± 0.14  **Group IV (water without F + Cd 50 mg/L):** 6; 4.8 ± 2.73  (N; mean ± SD) | |
| Zhang et al., 2013 | Water intake | | Blood from Sprague–Dawley rats | | Cd (μg/L)  **Group I (F 100 mg/L):** 6; 0.13 ± 0.09  **Group II (F 100 mg/L + Cd 50 mg/L):** 6; 2.78 ± 0.78*  (N; mean ± SD) | | Cd (μg/L)  **Group III (water without F and Cd):** 6; 0.13 ± 0.11  **Group IV (water without F + Cd 50 mg /L):** 6; 3.2 ± 0.67  (N; mean ± SD) | |
|  |  |  | Urinary from Sprague–Dawley rats | | Cd (μg/mmol creatinine)  **Group I (F 100 mg/L):** 6; 0.01 ± 0.02  **Group II (F 100 mg/L + Cd 50 mg/L):** 6; 0.38 ± 0.19*  (N; mean ± SD) | | Cd (μg/mmol creatinine)  **Group III (water without F and Cd):** 6; 0.02 ± 0.01  **Group IV (water without F + Cd 50 mg /L):** 6; 0.41 ± 0.22  (N; mean ± SD) | |
| Li et al., 2023 | Water intake | | Liver from Sprague-Dawley rats | | Cd (mg/Kg)  **Group I (F 15 mg/Kg + Cd 1 mg/Kg):** 6; 4.93 ± 0.23*^#^  **Group II (F 55 mg/Kg + Cd 1 mg/Kg):** 6; 2.89 ± 0.22*^#^  **Group III (F 75 mg/Kg + Cd 1 mg/Kg):** 6; 1.45 ± 0.09^#^  (N; mean ± SEM) | | Cd (mg/Kg)  **Group IV (water without F + Cd 1 mg/Kg):** 6; 7.15 ± 0.72  **Group V (water without F and Cd):** 6; 0.04 ± 0.01  (N; mean ± SEM) | |
|  |  |  | Kidney from Sprague-Dawley rats | | Cd mg/Kg (ppm)  **Group I (F 15 mg/Kg + Cd 1 mg/Kg):** 6; 7.41 ± 0.67*  **Group II (F 55 mg/Kg + Cd 1 mg/Kg):** 6; 7.32 ± 0.59*  **Group III (F 75 mg/Kg + Cd 1 mg/Kg):** 6; 3.23 ± 0.55*^#^  (N; mean ± SEM) | | Cd mg/Kg (ppm)  **Group IV (water without F + Cd 1 mg/Kg):** 6; 9.07 ± 1.01  **Group V (water without F and Cd):** 6; 0.02 ± 0.01  (N; mean ± SEM) | |
|  |  |  | Colon from Sprague-Dawley rats | | Cd mg/Kg (ppm)  **Group I (F 15 mg/Kg + Cd 1 mg/Kg):** 6; 0.50 ± 0.04*  **Group II (F 55 mg/Kg + Cd 1 mg/Kg):** 6; 0.42 ± 0.07*  **Group III (F 75 mg/Kg + Cd 1 mg/Kg):** 6; 0.27 ± 0.02^#^  (N; mean ± SEM) | | Cd mg/Kg (ppm_  **Group IV (water without F + Cd 1 mg/Kg):** 6; 0.52 ± 0.02  **Group V (water without F and Cd):** 6; 0.20 ± 0.05  (N; mean ± SEM) | |
| Li et al., 2024b | Water intake | | Colon from Sprague-Dawley rats | | Cd mg/Kg (ppm)  **Group I (F 15 mg/Kg + Cd 1 mg/Kg):** 6; 5.6 ± 0.7*^#^  **Group II (F 45 mg/Kg + Cd 1 mg/Kg):** 6; 5.58 ± 0.75*  **Group III (F 75 mg/Kg + Cd 1 mg/Kg):** 6; 9.37 ± 1.14*  (N; mean ± SEM) | | Cd mg/Kg (ppm)  **Group IV (Cd 1 mg/Kg):** 6; 11.4 ± 0.59  **Group V (water without F and Cd):** 6; 0.03 ± 0.01  (N; mean ± SEM) | |
| **MICE** | | | | | | | |  |
| Singh, 1982 | Water intake | Liver from Swiss albino mice | | Mn μg/g (ppm)  **Group I (F 10 ppm):** (8; 6.9 ± 1.3)  **Group II (F 25 ppm):** (8; 6.2 ± 1.2)  **Group III (F 50 ppm):** (8; 4.6 ± 0.57)*  **Group IV (F 100 ppm):** (8; 3.9 ± 0.47)*  **Group V (F 200 ppm):** (8; 3.6 ± 0.34)*  (N; mean ± SD) | | Mn μg/g (ppm)  **Water without F:** (8; 6.4 ± 1.2)  (N; mean ± SD) | |  |
|  |  | Kidney from Swiss albino mice | | Mn μg/g (ppm)  **Group I (F 10 ppm):** (8; 9.8 ± 1.4)  **Group II (F 25 ppm):** (8; 8.9 ± 1.4)  **Group III (F 50 ppm):** (8; 8.9 ± 1.5)  **Group IV (F 100 ppm):** (8; 6.9 ± 0.67)*  **Group V (F 200 ppm):** (8; 6.6 ± 1.0)*  (N; mean ± SD) | | Mn μg/g (ppm)  **Water without F:** (8; 9.7 ± 1.3)  (N; mean ± SD) | |  |
|  |  | Femur from Swiss albino mice | | Mn μg/g (ppm)  **Group I (F 10 ppm):** (8; 4.1 ± 0.64)  **Group II (F 25 ppm):** (8; 4.7 ± 0.73)*  **Group III (F 50 ppm):** (8; 5.6 ± 0.38)*  **Group IV (F 100 ppm):** (8; 5.9 ± 0.58)*  **Group V (F 200 ppm):** (8; 6.3 ± 0.57)*  (N; mean ± SD) | | Mn μg/g (ppm)  **Water without F:** (8; 3.8 ± 0.72)  (N; mean ± SD) | |  |
| Cooke et al., 1990 | Water intake | Liver from Wood mice | | Pb, Zn, and Cd mg/Kg (ppm)  **Group I (F 200 mg/L [ppm]):**  Pb (10; 1.43 to 3.47), Zn (10; 116 to 237), and Cd (10; 0.14 to 0.79)  **Group II (F 200 mg/L [ppm] + Pb 200 mg/L [ppm]):**  Pb (10; 3.91 to 7.94), Zn (10; 86 to 226), and Cd (10; 0.07 to 0.22)  **Group III (F 200 mg/L [ppm] + Pb 200 mg/L [ppm] + Zn 200 mg/L [ppm] + Cd 10 mg/L [ppm]):**  Pb (10; 2.39 to 10.10), Zn (10; 49.6 to 186), and Cd (10; 0.85 to 4.39)  (N; mean, 95 % confidence limits) | | Pb, Zn, and Cd mg/Kg (ppm)  **Group IV (water without F, Pb, Zn, and Cd):**  Pb (10; 1.36 to 4.55), Zn (10; 84.2 to 257), and Cd (10; 0.1 to 0.34)  **Group V (water without F + Pb 200 mg/L [ppm]):**  Pb (10; 1.59 to 6.30), Zn (10; 131 to 293), and Cd (10; 0.11 to 0.30)  **Group VI (water without F + Zn 200 mg/L [ppm]):**  Pb (10; 1.67 to 3.18), Zn (10; 47.7 to 169), and Cd (10; 3.02 to 6.13)  **Group VII (water without F + Cd 10 mg/L [ppm]):**  Pb (10; 1.13 to 2.46), Zn (10; 106 to 309), and Cd (10; 0.06 to 0.28)  **Group VIII (water without F + Zn 200 mg/L [ppm] + Cd 10 mg/L [ppm]):**  Pb (10; 1.18 to 6.08), Zn (10; 154 to 481), and Cd (10; 0.83 to 3.56)  (N; mean, 95 % confidence limits) | |  |
|  |  | Kidney from Wood mice | | Pb, Zn, and Cd mg/Kg (ppm)  **Group I (F 200 mg/L [ppm]):**  Pb (10; 0.93 to 3.32), Zn (10; 110 to 348), and Cd (10; 0.1 to 0.37)  **Group II (F 200 mg/L [ppm] + Pb 200 mg/L [ppm]):**  Pb (10; 9.81 to 21.8), Zn (10; 228 to 478), and Cd (10; 0.32 to 1.30)  **Group III (F 200 mg/L [ppm] + Pb 200 mg/L [ppm] + Zn 200 mg/L [ppm] + Cd 10 mg/L [ppm]):**  Pb (10; 8.73 to 28.50), Zn (10; 306 to 566), and Cd (10; 7.78 to 21.1)  (N; Mean, 95 % confidence limits) | | Pb, Zn, and Cd mg/Kg (ppm)  **Group IV (water without F, Pb, Zn, and Cd):**  Pb (10; 1.86 to 3.46), Zn (10; 94.1 to 206), and Cd (10; 0.12 to 0.68)  **Group V (water without F + Pb 200 mg/L [ppm]):**  Pb (10; 3.26 to 13.9), Zn (10; 85 to 278), and Cd (10; 0.1 to 0.81)  **Group VI (water without F + Zn 200 mg/L [ppm]):**  Pb (10; 1 to 4), Zn (10; 1 to 4), and Cd (10; 3.82 to 15.3)  **Group VII (water + Cd 10 mg/L [ppm]):**  Pb (10; 6.59 to 9.9), Zn (10; 448 to 604), and Cd (10; 0.2 to 0.88)  **Group VIII (water + Zn 200 mg/L [ppm] + Cd 10 mg/L [ppm]):**  Pb (10; 2.61 to 6.73), Zn (10; 265 to 550), and Cd (10; 1.74 to 9.36)  (N; mean, 95 % confidence limits) | |  |
|  |  | Femur from Wood mice | | Pb, Zn, and Cd mg/Kg (ppm)  **Group I (F 200 mg/L [ppm]):**  Pb (10; 3.57 to 9.65), Zn (10; 245 to 560), and Cd (10; 0.06 to 0.18)  **Group II (F 200 mg/L [ppm] + Pb 200 mg/L [ppm]):**  Pb (10; 38.8 to 98), Zn (10; 62.7 to 316), and Cd (10; 0.03 to 0.18)  **Group III (F 200 mg/L [ppm] + Pb 200 mg/L [ppm] + Zn 200 mg/L [ppm] + Cd 10 mg/L [ppm]):**  Pb (10; 64.8 to 185.3), Zn (10; 60.3 to 275), and Cd (10; 0.1 to 0.36)  (N; mean, 95 % confidence limits) | | Pb, Zn, and Cd (mg/Kg (ppm)  **Group IV (water without F, Pb, Zn, and Cd):**  Pb (10; 1.93 to 4.28), Zn (10; 106 to 271), and Cd (10; 0.02 to 0.12)  **Group V (water without F + Pb 200 mg/L [ppm]):**  Pb (10; 29.3 to 116.3), Zn (10; 57 to 223), and Cd (10; 0.02 to 0.13)  **Group VI (water without F + Zn 200 mg/L [ppm]):**  Pb (10; 1.47 to 3.84), Zn (10; 1.47 to 3.84), and Cd (10; 0.06 to 0.12)  **Group VII (water without F + Cd 10 mg/L [ppm]):**  Pb (10; 1.72 to 4.66), Zn (10; 1.37 to 299), Cd (10; 0.01 to 0.09)  **Group VIII (water without F + Zn 200 mg/L [ppm] + Cd 10 mg/L [ppm]):**  Pb (10; 1.63 to 7.61), Zn (10; 61.7 to 455), and Cd (10; 0.03 to 0.13)  (N; mean, 95 % confidence limits) | |  |
| Karademir, 2010 | Water intake | Serum from Swiss  albino mice | | Co, Mo, Cr, Mn (ng/mL), and Li μg/mL (ppm)  **Group I (F 10 mg/L [ppm]):**  Co (10; 0.32 ± 0.02), Mo (10; 1.44 ± 0.36), Cr (10; 0.37 ± 0.03), Mn (10; 0.76 ± 0.24), and Li (10; 4.74 ± 1.22)  **Group II (F 20 mg/L [ppm]):**  Co (10; 0.34 ± 0.04), Mo (10; 1.27 ± 0.36), Cr (10; 0.35 ± 0.02)*, Mn (10; 0.64 ± 0.22)*, and Li (10; 5.05 ± 1.35)  **Group III (F 30 mg/L [ppm]):**  Co (10; 0.37 ± 0.05), Mo (10; 0.85 ± 0.22)*, Cr (10; 0.35 ± 0.03)*, Mn (10; 0.5 ± 0.09)*, and Li (10; 5.39 ± 1.31)  **Group IV (F 40 mg/L [ppm]):**  Co (10; 0.40 ± 0.08)*, Mo (10; 0.52 ± 0.23)*, Cr (10; 0.34 ± 0.04)*, Mn (10; 0.43 ± 0.09)*, and Li (10; 5.35 ± 1.35)  (N; mean ± SE) | | Co, Mo, Cr, Mn (ng/mL), and Li μg/mL (ppm)  **Group V (Low-F tap water):**  Co (10; 0.33 ± 0.05), Mo (10; 1.51 ± 0.17), Cr (10; 0.39 ± 0.04), Mn (10; 0.96 ± 0.37), and Li (10; 5.41 ± 1.46)  (N; mean ± SE) | |  |
| Dey Bhowmi, 2020 | Water intake | Bone from Swiss albino mice | | K, Ca, Fe, Zn, Mg, Mn, Se, and Ni μg/g (ppm)  **Group I (NaF 15 ppm drinking water for 4 months):**  K (9; 93.1 ± 0.73)*, Ca (9; 86.2 ± 4.19)*, Fe (9; 94.4 ± 2.49), Zn (9; 87.2 ± 2.24)*, Mg (9; 105.5 ± 4.31)*, Mn (9; 76.8 ± 4.01)*, Se (9; 103 ± 4.0), and Ni (9; 56.3 ± 3.29)*  **Group II (NaF 15 ppm for 8 months):**  K (9; 95.1 ± 0.88)*, Ca (9; 76.1 ± 2.72)*, Fe (9; 147 ± 7.45)*, Zn (9; 86.5 ± 2.24)*, Mg (9; 108 ± 5.96)*, Mn (9; 76.8 ± 4.01)*, Se (9; 102 ± 3.2), and Ni (9; 36.6 ± 4.39)*  **Group III (orally treated with NaF 15 ppm for 4 months, thereafter received only drinking water for next 4 months):**  K (9; 95.9 ± 0.69)*, Ca (9; 93.6 ± 1.94)*, Fe (9; 172 ± 13.05)*, Zn (9; 91.7 ± 3.73)*, Mg (9; 103 ± 3.09), Mn (9; 87.2 ± 5.6)*, Se (9; 104 ± 3.2)*, and Ni (9; 59.6 ± 3.29)*  **Group IV (orally treated with NaF 15 ppm for 4 months, thereafter received only drinking water supplemented with calcium and vitamin D [2.5-g calcium/Kg diet and 1000 IU vitamin D/Kg diet] for next 4 months):**  K (9; 97.9 ± 0.92)*, Ca (9; 108.3 ± 2.72)*, Fe (9; 90.7 ± 7.45), Zn (9; 96.1 ± 7.46), Mg (9; 98.6 ± 0.99)*, Mn (9; 86.4 ± 8.0)*, Se (9; 84 ± 7.2)*, and Ni (9; 98.4 ± 5.12)  **Group V (NaF 15 ppm drinking water as well as supplemented with calcium and vitamin D for 4 months):**  K (9; 95.3 ± 0.88)*, Ca (9; 107.6 ± 1.94)*, Fe (9; 91.3 ± 4.35), Zn (9; 84.2 ± 8.2)*, Mg (9; 97.7 ± 2.1)*, Mn (9; 74.4 ± 10.40)*, Se (9; 102 ± 3.0), and Ni (9; 95.8 ± 3.66)  (N; mean ± SEM) | | K, Ca, Fe, Zn, Mg, Mn, Se, and Ni μg/g (ppm)  **Group VI (water with F < 0.5 ppm):**  K (9; 100), Ca (9; 100), Fe (9; 99.4), Zn (9; 100), Mg (9; 100), Mn (9; 100 ± 2.40), Se (9; 102), and Ni (9; 100.0 ± 2.07)  (N; mean ± SEM) | |  |
| Pena et al., 2023 | Water intake | Kidney from C57BL/6 mice | | iAs, MAs, DMAs, and Sum As species (ng/g)  **Group I (F 50 mg/L [ppm]):**  iAs (8; 0.14 ± 0.01), MAs (8; ≤ 0.13), DMAs (8; 1.49 ± 0.27), and Sum As species (8; 1.77 ± 0.27)  **Group II (F 50 mg/L [ppm] + As 1 mg/L [ppm]):**  iAs (8; 3.25 ± 0.24)^#^, MAs (8; 0.75 ± 0.16)^#^, DMAs (8; 20.24 ± 1.28)^#^, and Sum As species (8; 23.64 ± 1.52)^#^  (N; mean ± SEM) | | iAs, MAs, DMAs, and Sum As species (ng/g)  **Group III (water without F):**  iAs (8; 0.25 ± 0.01), MAs (8; ≤ 0.13), DMAs (8; 0.63 ± 0.03), and Sum As species (8; 1.03 ± 0.03)  **Group IV (water without F + AS 1 mg/L [ppm]):**  iAs (8; 4.76 ± 0.68), MAs (8; 1.47 ± 0.24), DMAs (8; 40.32 ± 5.61), and Sum As species (8; 46.55 ± 6.40)  (N; mean ± SEM) | |  |
|  |  | Liver from C57BL/6 mice | | iAs, MAs, DMAs, and Sum As species (ng/g)  **Group I (F 50 mg/L [ppm]):**  iAs (8; 0.16 ± 0.03), MAs (8; ≤ 0.13), DMAs (8; 2.03 ± 0.22), and Sum As species (8; 2.34 ± 0.23)  **Group II (F 50 mg/L [ppm] + As 1 mg/L [ppm]):**  iAs (8; 3.59 ± 0.49)^#^, MAs (8; 3.01 ± 0.51)^#^, DMAs (8; 21.6 ± 1.52)^#^, and Sum As species (8; 28.2 ± 2.02)^#^  (N; mean ± SEM) | | iAs, MAs, DMAs, and Sum As species (ng/g)  **Group III (water without F):**  iAs (8; 1.10 ± 0.09), MAs (8; 0.22 ± 0.02), DMAs (8; 0.48 ± 0.04), and Sum As species (8; 1.80 ± 0.12)  **Group IV (water without F + AS 1 mg/L [ppm]):**  iAs (8; 9.26 ± 1.96), MAs (8; 7.3 ± 1.26), DMAs (8; 40 ± 3.47), and Sum As species (8; 56.5 ± 6.29)  (N; mean ± SEM) | |  |
|  |  | Lung from C57BL/6 mice | | iAs, MAs, DMAs, and Sum As species (ng/g)  **Group I (F 50 mg/L [ppm]):**  iAs (8; 0.16 ± 0.17), MAs (8; 0.16 ± 0.02), DMAs (8; 3.37 ± 0.77), and Sum As species (8; 4.11 ± 0.85)  **Group II (F 50 mg/L [ppm] + As 1 mg/L [ppm]):**  iAs (8; 1.32 ± 0.28), MAs (8; 0.81 ± 0.17), DMAs (8; 51.9 ± 6.23), and Sum As species (8; 54 ± 6.49)  (N; mean ± SEM) | | iAs, MAs, DMAs, and Sum As species (ng/g)  **Group III (water without F):**  iAs (8; 0.16 ± 0.02), MAs (8; 0.17 ± 0.01), DMAs (8; 0.59 ± 0.03), and Sum As species (8; 0.91 ± 0.05)  **Group IV (water without F + AS 1 mg/L [ppm]):**  iAs (8; 2.05 ± 0.26), MAs (8; 1.25 ± 0.15), DMAs (8; 68.7 ± 5.80), Sum As species (8; 71.9 ± 5.99)  (N; mean ± SEM) | |  |
|  |  | Urinary Bladder from C57BL/6 mice | | iAs, MAs, DMAs, and Sum As species (ng/g)  **Group I (F 50 mg/L [ppm]):**  iAs (8; ≤ 0.13), MAs (8; 10.2 ± 4.58)^#^, DMAs (8; 12.2 ± 5.28), and Sum As species (8; 25.4 ± 12)  **Group II (F 50 mg/L [ppm] + As 1 mg/L [ppm]):**  iAs (8; 7.51 ± 2.9), MAs (8; 0.14 ± 0.01)^#^, DMAs (8; 344 ± 76.6), and Sum As species (8; 368 ± 96.4)  (N; mean ± SEM) | | iAs, MAs, DMAs, and Sum As species (ng/g)  **Group III (water without F):**  iAs (8; ≤ 0.13), MAs (8; 9.62 ± 4.13), DMAs (8; 11.8 ± 5.25), and Sum As species (8; 20.01 ± 8.13)  **Group IV (water without F + AS 1 mg/L [ppm]):**  iAs (8; 10.9 ± 3.64), MAs (8; 1.62 ± 0.75), DMAs (8; 602 ± 114), and Sum As species (8; 644 ± 139)  (N; mean ± SEM) | |  |
|  |  | Urine from C57BL/6 mice | | iAs, MAs, DMAs, and Sum As species (ng/g)  **Group I (F 50 mg/L [ppm]):**  iAs (4; 1.67 ± 0.56), MAs (4; ≤ 0.13), DMAs (4; 29.6 ± 6.26), and Sum As species (4; 31.4 ± 7.18)  Group II (F 50 mg/L [ppm] + As 1 mg/L [ppm]):  iAs (4; 32 ± 5.99)^#^, MAs (4; 3.99 ± 0.4)^#^, DMAs (4; 370 ± 35.5)^#^, and Sum As species (4; 405.8 ± 35.6)  (N; mean ± SEM) | | iAs, MAs, DMAs, and Sum As species (ng/g)  **Group III (water without F):**  iAs (4; 1.8 ± 0.67), MAs (4; ≤ 0.13), DMAs (4; 35 ± 6.54), and Sum As species (4; 37.11 ± 6.32)  **Group IV (water without F + AS 1 mg/L [ppm]):**  iAs (4; 20.61 ± 1.9), MAs (4; 2.56 ± 0.43), DMAs (4; 478.2 ± 76.11), and Sum As species (4; 501.4 ± 77.4)  (N; mean ± SEM) | |  |
| Hu et al., 2024 | Water intake | Bone from wild-type mice | | Ca (mmol/gprot)  **NaF 100 mg/L (ppm):** 6; 0.15 ± 0.01*  (N; mean ± SEM) | | Ca (mmol/gprot)  **Water without F:** 6; 0.18 ± 0.01  (N; mean ± SEM) | |  |
|  |  | Bone from Parkin -/- C57BL/6 mice | | Ca (mmol/gprot)  **NaF 100 mg/L (ppm):** 6; 0.15 ± 0.01  (N; mean ± SEM) | | Ca (mmol/gprot)  **Water without F:** 6; 0.18 ± 0.01  (N; mean ± SEM) | |  |
|  |  | Bone from C57BL/6 mice | | Ca (mmol/gprot)  **Group I (NaF 100 mg/L [ppm]):** 6; 0.10 ± 0.01*  **Group II (NaF 100 mg/L [ppm] + 1% CaCO_3_):** 6; 0.10 ± 0.01*  (N; mean ± SEM) | | Ca (mmol/gprot)  **Group III (water with 1 % CaCO_3_):** 6; 0.16 ± 0.01  **Group IV (water without F):** 6; 0.14 ± 0.01  (N; mean ± SEM) | |  |
| Li et al., 2024a | Daily gavage | Testis from IL-17A knockout C57BL/6J mice and wild type (WT) mice | | Fe (μmol/g protein)  **WT (NaF 24 mg/Kg [ppm]):** 5; 6.8 ± 2.26  **IL-17A (NaF 24 mg/Kg [ppm]):** 5; 5.9 ± 1.58  (N; mean ± SEM) | | Fe (μmol/g protein)  **WT (saline 0.1 mL/10 g):** 5; 5.46 ± 0.77  **IL-17A (saline 0.1 mL/10 g):** 5; 5.49 ± 0.95  (N; mean ± SEM) | |  |
| **PIGS** | | | | | | | |  |
| Tao et al., 2005 | Diet treatments added to the corn-soybean basal diet | Liver from pigs | | Fe, Zn, Cu, and Mn μg/g (ppm)  **Group I (corn-soybean basal diet [F 39.75 mg/Kg {ppm}] + F 50 mg/Kg [ppm]):**  Fe (2; 61.1 ± 2.53), Zn (2; 46.8 ± 1.38), Cu (2; 8.94 ± 0.41), and Mn (2; 1.26 ± 0.08)  **Group II (corn-soybean basal diet [F 39.75 mg/Kg {ppm}] + F 100 mg/Kg [ppm]):**  Fe (2; 49.7 ± 2.53)*, Zn (2; 41.2 ± 1.38)*, Cu (2; 7.49 ± 0.41)*, and Mn (2; 0.1 ± 0.08)*  **Group III (corn-soybean basal diet [F 39.75 mg/Kg {ppm}] + F 150 mg/Kg [ppm]):**  Fe (2; 39.6 ± 2.53)*, Zn (2; 35.8 ± 1.38)*, Cu (2; 4.51 ± 0.41)*, and Mn (2; 0.66 ± 0.08)*  (N; mean ± SEM) | | Fe, Zn, Cu, and Mn μg/g (ppm)  **Group IV (Corn-soybean basal diet [F 39.75 mg/Kg {ppm}] without added F):**  Fe (2; 65.8 ± 2.53), Zn (2; 46 ± 1.38), Cu (2; 9.46 ± 0.41), and Mn (2; 1.42 ± 0.08)  (N; mean ± SEM) | |  |
|  |  | Renal cortex from pigs | | Fe, Zn, Cu, and Mn μg/g (ppm)  **Group I (corn-soybean basal diet [F 39.75 mg/Kg {ppm}] + F 50 mg/Kg [ppm]):**  Fe (2; 26.9 ± 0.89), Zn (2; 21.2 ± 0.95), Cu (2; 8.7 ± 0.32), and Mn (2; 0.9 ± 0.04)  **Group II (corn-soybean basal diet [F 39.75 mg/Kg {ppm}] + F 100 mg/Kg [ppm]):**  Fe (2; 49.7 ± 2.53)*, Zn (2; 25.6 ± 0.95)*, Cu (2; 8.56 ± 0.32)*, and Mn (2; 0.76 ± 0.04)*  **Group III (corn-soybean basal diet [F 39.75 mg/Kg {ppm}] + F 150 mg/Kg [ppm]):**  Fe (2; 39.6 ± 2.53)*, Zn (2; 29.1 ± 0.95)*, Cu (2; 7.24 ± 0.32)*, and Mn (2; 0.46 ± 0.04)*  (N; mean ± SEM) | | Fe, Zn, Cu, and Mn μg/g (ppm)  **Group IV (Corn-soybean basal diet [F 39.75 mg/Kg {ppm}] without added F):**  Fe (2; 27.3 ± 0.89), Zn (2; 21.4 ± 0.95), Cu (2; 9.51 ± 0.32), and Mn (2; 0.9 ± 0.04)  (N; mean ± SEM) | |  |
|  |  | Heart from pigs | | Fe, Zn, Cu, and Mn μg/g (ppm)  **Group I (corn-soybean basal diet [F 39.75 mg/Kg {ppm}] + F 50 mg/Kg [ppm]):**  Fe (2; 28.3 ± 0.96), Zn (2; 16.1 ± 0.47), Cu (2; 2.6 ± 0.16), and Mn (2; 1.11 ± 0.04)  **Group II (corn-soybean basal diet [F 39.75 mg/Kg {ppm}] + F 100 mg/Kg [ppm]):**  Fe (2; 26.6 ± 0.96), Zn (2; 16.5 ± 0.47), Cu (2; 1.77 ± 0.16)*, and Mn (2; 0.88 ± 0.04)*  **Group III (corn-soybean basal diet [F 39.75 mg/Kg {ppm}] + F 150 mg/Kg [ppm]):**  Fe (2; 26.1 ± 0.96), Zn (2; 16 ± 0.47), Cu (2; 1.32 ± 0.16)*, and Mn (2; 0.62 ± 0.04)*  (N; mean ± SEM) | | Fe, Zn, Cu, and Mn μg/g (ppm)  **Group IV (Corn-soybean basal diet [F 39.75 mg/Kg {ppm}] without added F):**  Fe (2; 28.2 ± 0.96), Zn (2; 16.2 ± 0.47), Cu (2; 2.52 ± 0.16), and Mn (2; 1.04 ± 0.04)  (N; mean ± SEM) | |  |
|  |  | Pancreas from pigs | | Fe, Zn, Cu, and Mn μg/g (ppm)  **Group I (corn-soybean basal diet [F 39.75 mg/Kg {ppm}] + F 50 mg/Kg [ppm]):**  Fe (2; 29.1 ± 0.91), Zn (2; 28.1 ± 0.99), Cu (2; 6.14 ± 0.18), and Mn (2; 1.48 ± 0.05)  **Group II (corn-soybean basal diet [F 39.75 mg/Kg {ppm}] + F 100 mg/Kg [ppm]):**  Fe (2; 28 ± 0.91), Zn (2; 25 ± 0.99)*, Cu (2; 6.06 ± 0.18), Mn (2; 1.07 ± 0.05)*  **Group III (corn-soybean basal diet [F 39.75 mg/Kg {ppm}] + F 150 mg/Kg [ppm]):**  Fe (2; 27.2 ± 0.91), Zn (2; 22.1 ± 0.99)*, Cu (2; 6.09 ± 0.18), and Mn (2; 0.72 ± 0.05)*  (N; mean ± SEM) | | Fe, Zn, Cu, and Mn μg/g (ppm)  **Group IV (Corn-soybean basal diet [F 39.75 mg/Kg {ppm}] without added F):**  Fe (2; 28.5 ± 0.91), Zn (2; 28.9 ± 0.99), Cu (2; 6.1 ± 0.18), and Mn (2; 1.57 ± 0.05)  (N; mean ± SEM) | |  |
|  |  | Spleen from pigs | | Fe, Zn, Cu, and Mn μg/g (ppm)  **Group I (corn-soybean basal diet [F 39.75 mg/Kg {ppm}] + F 50 mg/Kg [ppm]):**  Fe (2; 48.9 ± 1.98), Zn (2; 20.4 ± 0.7), Cu (2; 9.4 ± 0.36), and Mn (2; 0.81 ± 0.03)  **Group II (corn-soybean basal diet [F 39.75 mg/Kg {ppm}] + F 100 mg/Kg [ppm]):**  Fe (2; 39.7 ± 1.98)*, Zn (2; 20.6 ± 0.7), Cu (2; 8.12 ± 0.36)*, and Mn (2; 0.74 ± 0.03)  **Group III (corn-soybean basal diet [F 39.75 mg/Kg {ppm}] + F 150 mg/Kg [ppm]):**  Fe (2; 26.6 ± 1.98)*, Zn (2; 19.7 ± 0.7), Cu (2; 5.18 ± 0.36)*, and Mn (2; 0.75 ± 0.03)  (N; mean ± SEM) | | Fe, Zn, Cu, and Mn μg/g (ppm)  **Group IV (Corn-soybean basal diet [F 39.75 mg/Kg {ppm}] without added F):**  Fe (2; 52.8 ± 1.98), Zn (2; 20 ± 0.7), Cu (2; 10.4 ± 0.36), and Mn (2; 0.77 ± 0.03)  (N; mean ± SEM) | |  |
|  |  | Brain from pigs | | Fe, Zn, Cu, and Mn μg/g (ppm)  **Group I (corn-soybean basal diet [F 39.75 mg/Kg {ppm}] + F 50 mg/Kg) [ppm]:**  Fe (2; 27.6 ± 0.98), Zn (2; 8.36 ± 0.29), Cu (2; 7.72 ± 0.27), and Mn (2; 0.67 ± 0.02)  **Group II (corn-soybean basal diet [F 39.75 mg/Kg {ppm}] + F 100 mg/Kg [ppm]):**  Fe (2; 23.6 ± 0.98)*, Zn (2; 7.95 ± 0.29), Cu (2; 6.83 ± 0.27)*, and Mn (2; 0.49 ± 0.02)*  **Group III (corn-soybean basal diet [F 39.75 mg/Kg {ppm}] + F 150 mg/Kg [ppm]):**  Fe (2; 22.3 ± 0.98)*, Zn (2; 8.08 ± 0.29), Cu (2; 5.97 ± 0.27)*, and Mn (2; 0.46 ± 0.02)*  (N; mean ± SEM) | | Fe, Zn, Cu, and Mn μg/g (ppm)  **Group IV (Corn-soybean basal diet [F 39.75 mg/Kg {ppm}] without added F):**  Fe (2; 26.6 ± 0.98), Zn (2; 8.22 ± 0.29), Cu (2; 8.04 ± 0.27), and Mn (2; 0.66 ± 0.02)  (N; mean ± SEM) | |  |
|  |  | Bone from pigs | | Fe, Zn, Cu, and Mn μg/g (ppm)  **Group I (corn-soybean basal diet [F 39.75 mg/Kg {ppm}] + F 50 mg/Kg [ppm]):**  Fe (2; 14.4 ± 0.57), Zn (2; 47.4 ± 1.5), Cu (2; 5.12 ± 0.18), and Mn (2; 1.62 ± 0.06)  **Group II (corn-soybean basal diet [F 39.75 mg/Kg {ppm}] + F 100 mg/Kg [ppm]):**  Fe (2; 10.1 ± 0.57)*, Zn (2; 43 ± 1.5)*, Cu (2; 5.21 ± 0.18), and Mn (2; 1.31 ± 0.06)*  **Group III (corn-soybean basal diet [F 39.75 mg/Kg {ppm}] + F 150 mg/Kg [ppm]):**  Fe (2; 8.3 ± 0.57)*, Zn (2; 37.5 ± 1.5)*, Cu (2; 5.08 ± 0.18), and Mn (2; 0.94 ± 0.06)*  (N; mean ± SEM) | | Fe, Zn, Cu, and Mn μg/g (ppm)  **Group IV (Corn-soybean basal diet [F 39.75 mg/Kg {ppm}] without added F):**  Fe (2; 15.2 ± 0.57), Zn (2; 48.2 ± 1.5), Cu (2; 5.13 ± 0.18), and Mn (2; 1.59 ± 0.06)  (N; mean ± SEM) | |  |
|  |  | Serum from pigs | | Fe, Zn, Cu, and Mn (μg/mL)  **Group I (corn-soybean basal diet [F 39.75 mg/Kg {ppm}] + F 50 mg/Kg [ppm]):**  Fe (2; 52.3 ± 1.41), Zn (2; 12.4 ± 0.36), Cu (2; 8.51 ± 0.23), and Mn (2; 4.49 ± 0.14)  **Group II (corn-soybean basal diet (F 39.75 mg/Kg {ppm}] + F 100 mg/Kg [ppm]):**  Fe (2; 44.6 ± 1.41), Zn (2; 11.31 ± 0.36)*, Cu (2; 7.34 ± 0.23)*, and Mn (2; 3.69 ± 0.14)*  **Group III (corn-soybean basal diet [F 39.75 mg/Kg {ppm}] + F 150 mg/Kg [ppm]):**  Fe (2; 32.8 ± 1.41), Zn (2; 9.37 ± 0.36)*, Cu (2; 5.83 ± 0.23)*, and Mn (2; 2.81 ± 0.14)*  (N; mean ± SEM) | | Fe, Zn, Cu, and Mn (μg/mL)  **Group IV (Corn-soybean basal diet [F 39.75 mg/Kg {ppm}] without added F):**  Fe (2; 55.1 ± 1.41), Zn (2; 12. ± 0.36), Cu (2; 8.91 ± 0.23), and Mn (2; 4.45 ± 0.14)  (N; mean ± SEM) | |  |
| Khandare et al., 2015 | Water intake | Serum from Guinea pigs (*Cavia porcellus*) | | Sr (μg/dL)  **Group I (F 25 mg/L [ppm]):**  2 months (6; 21.7), 4 months (6; 16.3), and 6 months (6; 27.2)  **Group II (F 25 mg/L [ppm] + Sr 0.59 %):**  2 months (6; 796 ± 125), 4 months (6; 1166 ± 550), and 6 months (6; 131 ± 115)*  Ca (mg/dL)  **Group I (F 25 mg/L [ppm]):**  60 days (6; 17.9 ± 2.79), and 120 days (6; 18.2 ± 1.02)  **Group II (F 25 mg/L [ppm] + Sr 0.59 %):**  60 days (6; 16 ± 2.01), and 120 days (6; 13.9 ± 3.33)*  (N; mean ± SD) | | Sr (μg/dL)  **Group III (water without F and Sr):**  2 months (6; 21.7 ± 0.0), 4 months (6; 10.8 ± 0.0), and 6 months (6; 21.7 ± 0.0)  **Group IV (water without F + Sr 0.59 %):**  2 months (6; 1117 ± 169), 4 months (6; 1204 ± 637), and 6 months (6; 518 ± 556)  Ca (mg/dL)  **Group III (water without F and Sr):**  60 days (6; 18.5 ± 2.02), and 120 days (6; 18.8 ± 2.32)  **Group IV (water without F + Sr 0.59 %):**  60 days (6; 15.4 ± 3.03), and 120 days (6; 16.8 ± 3.18)  (N; mean ± SD) | |  |
| **CATTLE** | | | | | | | |  |
| Ranjan et al., 2008 | Cattle exposed to endemic fluoride pollution with clinical lesions suggestive of chronic fluoride toxicity and healthy catlle | Blood from cattle | | Mg, Zn, Cu, Co, and Mn μg/mL (ppm)  Mg (270; 21.4 ± 0.09)*, Zn (270; 1.82 ± 0.1)*, Cu (270; 0.49 ± 0.02)*, Co (270; 0.54 ± 0.05)*, and Mn (270; 0.10 ± 0.01)*  (N; mean ± SE) | | Mg, Zn, Cu, Co, and Mn μg/mL (ppm)  Mg (270; 16.2 ± 0.08), Zn (270; 2.17 ± 0.13), Cu (270; 0.63 ± 0.03), Co (270; 0.36 ± 0.01), and Mn (270; 0.21 ± 0.03)  (N; mean ± SE) | |  |
|  |  | Serum from cattle | | Ca (μg/dL)  270; 6.62 ± 0.43*  (N; mean ± SE) | | Ca (μg/dL)  270; 11.8 ± 0.39  (N; mean ± SE) | |  |
| Chhabra et al., 2012 | In vivo  Cattle of villages of the district Muktsar, a brackish water zone, of the Punjab state | Plasma from cattle | | **Cattle from villages with a water fluoride concentration of more than 1 ppm and cattle showing signs of dental lesions or lameness were considered enzootic for fluorosis**  Ca (mg/dL), Mg, Mo μg/g (ppm), Fe, Zn, Cu, and Mn (μmol/L)  Ca (103; 9.23 ± 0.18)*, Mg (103; 2.48 ± 0.05)*, Fe (103; 132 ± 11.69)*, Zn (103; 21 ± 0.92)*, Cu (103; 8.41 ± 0.38)*, Mn (103; 1.31 ± 0.27), and Mo (103; 0.15 ± 0.01)  (N; mean ± SE) | | **Cattle from villages with a water fluoride concentration of less than 1 ppm and cattle with no clinical signs were considered fluorosis-free**  Ca (mg/dL), Mg, Mo μg/g (ppm), Fe, Zn, Cu, and Mn (μmol/L)  Ca (103; 9.76 ± 0.18), Mg (103; 2.91 ± 0.08), Fe (103; 99.05 ± 7.37), Zn (103; 27 ± 1.44), Cu (103; 10.7 ± 0.35), Mn (103; 1.03 ± 0.04), and Mo (103; 0.15 ± 0.04),  (N; mean ± SE) | |  |
| **RABBITS** | | | | | | | |  |
| Khandare et al., 2011 | Daily orally administration | Urine from New Zealand white rabbits | | Mg (mg/24h), Zn, and Cu (μg/24h)  **Group I (F 10 mg/Kg/day):**  Ca (6; 32.5 ± 22.2), Zn (6; 95.9 ± 62.6), Mg (6;11.8 ± 5.94), and Cu (6; 3.6 ± 0.96)  **Group II (F 10 mg/Kg/day + Mg 20 mg/Kg):**  Ca (6; 41.8 ± 24.1), Zn (6; 100 ± 51), Mg (6; 18.7 ±14.4), and Cu (6; 3.8 ± 0.73)  (N; mean ± SD) | | Mg (mg/24h), Zn, and Cu (μg/24h)  **Group III (water without F and Mg):**  Ca (6; 48.6 ± 37.2), Zn (6; 83.3 ± 49.2), Mg (6; 19.1 ± 16.6), and Cu (6; 5 ± 1.89)  (N; mean ± SD) | |  |
|  |  | Serum from New Zealand white rabbits | | Ca, Mg, Zn, and Cu (mg/dL)  **Group I (F 10 mg/Kg/day):**  Ca (6; 9.8 ± 1.53)*, Zn (6; 0.16 ± 0.04), Mg (6; 3.3 ± 3.13), and Cu (6; 0.12 ± 0.01)  **Group II (F 10 mg/Kg/day + Mg 20 mg/Kg):**  Ca: (6; 12.6 ± 0.8), Zn (6; 0.19 ± 0.02), Mg (6; 3.1 ± 0.45), and Cu (6; 0.13 ± 0.03)  (N; mean ± SD) | | Ca, Mg, Zn, and Cu (mg/dL)  **Group III (water without F and Mg):**  Ca (6; 12.4 ± 1.32), Zn (6; 0.24 ± 0.04), Mg (6; 3.0 ± 0.34), and Cu (6; 0.17 ± 0.06)  (N; mean ± SD) | |  |
| Ranjan et al., 2011 | Water intake | Liver from New Zealand white rabbits | | Zn, Cu, Mn, and Co μg/g (ppm)  **Group I (NaF 50 μg/mL [ppm]):**  Zn (6; 7.06 ± 0.26)*, Cu (6; 2.29 ± 0.16), Mn (6; 1.77 ± 0.13)*, and Co (6; 0.68 ± 0.07)*  **Group II (NaF 100 μg/mL [ppm]):**  Zn (6; 13 ± 0.91), Cu (6; 2.86 ± 0.33), Mn (6; 2.65 ± 0.55), and Co (6; 0.73 ± 0.20)*  **Group III (NaF 200 μg/mL [ppm]):**  Zn (6; 12.65 ± 0.58), Cu (6; 2.51 ± 0.26), Mn (6; 2.78 ± 0.63), and Co (6; 1.33 ± 0.15)  (N; mean ± SEM) | | Zn, Cu, Mn, and Co μg/g (ppm)  **Group IV (water without F):**  Zn (6; 14.58 ± 0.66), Cu (6; 2.7 ± 0.12), Mn (6; 3.66 ± 0.43), and Co (6; 1.34 ± 0.11)  (N; mean ± SEM) | |  |
|  |  | Kidney from New Zealand white rabbits | | Zn, Cu, Mn, and Co μg/g (ppm)  **Group I (NaF 50 μg/mL [ppm]):**  Zn (6; 7.43 ± 0.36)*, Cu (6; 1.96 ± 0.07), Mn (6; 1.60 ± 0.25), and Co (6; 0.83 ± 0.06)*  **Group II (NaF 100 μg/mL [ppm]):**  Zn (6; 10.5 ± 0.66), Cu (6; 1.56 ± 0.07), Mn (6; 2.08 ± 0.32), and Co (6; 1.12 ± 0.13)*  **Group III (NaF 200 μg/mL [ppm]):**  Zn (6; 11 ± 0.62), Cu (6; 2.47 ± 0.45), Mn (6; 1.87 ± 0.15), and Co (6; 1.60 ± 0.16)*  (N; mean ± SEM) | | Zn, Cu, Mn, and Co μg/g (ppm)  **Group IV (water without F):**  Zn (6; 11.3 ± 0.38), Cu (6; 2.3 ± 0.09), Mn (6; 2.23 ± 0.29), and Co (6; 1.38 ± 0.09)  (N; mean ± SEM) | |  |
|  |  | Muscle from New Zealand white rabbits | | Zn, Cu, Mn, and Co μg/g (ppm)  **Group I (NaF 50 μg/mL [ppm]):**  Zn (6; 3.88 ± 0.28), Cu (6; 0.33 ± 0.05)*, Mn (6; 1.4 ± 0.41), and Co (6; 0.71 ± 0.07)*  **Group II (NaF 100 μg/mL [ppm]):**  Zn (6; 4.01 ± 0.61), Cu (6; 0.23 ± 0.08)*, Mn (6; 2.14 ± 0.54), and Co (6; 1.13 ± 0.08)  **Group III (NaF 200 μg/mL [ppm]):**  Zn (6; 4.68 ± 0.37), Cu (6; 0.21 ± 0.07)*, Mn (6; 1.63 ± 0.14), and Co (6; 1.26 ± 0.17)  (N; mean ± SEM) | | Zn, Cu, Mn, and Co μg/g (ppm)  **Group IV (water without F):**  Zn (6; 5.23 ± 0.32), Cu (6; 0.44 ± 0.03), Mn (6; 2.1 ± 0.12), and Co (6; 1.4 ± 0.16)  (N; mean ± SEM) | |  |
|  |  | Heart from New Zealand white rabbits | | Zn, Cu, Mn, and Co μg/g (ppm)  **Group I (NaF 50 μg/mL [ppm]):**  Zn (6; 6.27 ± 0.17)*, Cu (6; 2.46 ± 0.4)*, Mn (6; 0.38 ± 0.05)*, and Co (6; 0.72 ± 0.07)*  **Group II (NaF 100 μg/mL [ppm]):**  Zn (6; 9.03 ± 0.43), Cu (6; 2.65 ± 0.2)*, Mn (6; 2.04 ± 0.38), and Co (6; 1.18 ± 0.29)*  **Group III (NaF 200 μg/mL [ppm]):**  Zn (6; 9 ± 0.19), Cu (6; 2.58 ± 0.18)*, Mn (6; 2.17 ± 0.15), and Co (6; 1.76 ± 0.18)  (N; mean ± SEM) | | Zn, Cu, Mn, and Co μg/g (ppm)  **Group IV (water without F):**  Zn (6; 10.4 ± 0.51), Cu (6; 3.46 ± 0.1), Mn (6; 2.17 ± 0.58), and Co (6; 1.8 ± 0.19)  (N; mean ± SEM) | |  |
|  |  | Lung from New Zealand white rabbits | | Zn, Cu, Mn, and Co μg/g (ppm)  **Group I (NaF 50 μg/mL [ppm]):**  Zn (6; 6.32 ± 0.48)*, Cu (6; 1.16 ± 0.13)*, Mn (6; 0.42 ± 0.01)*, and Co (6; 0.88 ± 0.08)*,  **Group II (NaF 100 μg/mL [ppm]):**  Zn (6; 10.3 ± 0.47), Cu (6; 0.79 ± 0.1)*, Mn (6; 1.76 ± 0.22), and Co (6; 1.45 ± 0.27)  **Group III (NaF 200 μg/mL [ppm]):**  Zn (6; 9.53 ± 0.27), Cu (6; 1.06 ± 0.08)*, Mn (6; 2.01 ± 0.48), and Co (6; 1.54 ± 0.22)  (N; mean ± SEM) | | Zn, Cu, Mn, and Co μg/g (ppm)  **Group IV (water without F):**  Zn (6; 9.06 ± 0.53), Cu (6; 1.37 ± 0.04), Mn (6; 2.34 ± 0.55), and Co (6; 1.52 ± 0.14),  (N; mean ± SEM) | |  |
| **SHEEP** | | | | | | | |  |
| Aydin et al., 2014 | Akkaraman sheep with and without fluorosis | Bone from Akkaraman sheeps | | **Fluorotic sheep**  Ca (mg/g), Mg, Zn, Cu, and Mn μg/g (ppm)  Ca (10; 166 ± 11.2), Mg (10; 2672 ± 232), Zn (10; 33.2 ± 6.41), Cu (10; 2.17 ± 0.35)*, and Mn (10; 0.48 ± 0.11)  (N; mean ± SD) | | **Non fluorotic sheep**  Ca (mg/g), Mg, Zn, Cu, and Mn μg/g (ppm)  Ca (10; 156.2 ± 16.2), Mg (10; 2799 ± 222), Zn (10; 32.5 ± 4.13), Cu (10; 4.08 ± 0.87), and Mn (10; 0.44 ± 0.04)  (N; mean ± SD) | |  |
|  |  | Tooth from Akkaraman sheeps | | **Fluorotic sheep**  Ca (mg/g), Mg, Zn, Cu, and Mn μg/g (ppm)  Ca (10; 175 ± 11.9)*, Mg (10; 3237 ± 367.52)*, Zn (10; 16.6 ± 2.3)*, Cu (10; 0.36 ± 0.08), and Mn (10; 0.15 ± 0.03)*  (N; mean ± SD) | | **Non Fluorotic sheep**  Ca (mg/g), Mg, Zn, Cu, and Mn μg/g (ppm)  Ca (10; 149.4 ± 6.16), Mg (10; 4768 ± 406), Zn (10; 20.3 ± 1.67), Cu (10; 2.23 ± 0.36), and Mn (10; 4.19 ± 0.97)  (N; mean ± SD) | |  |
|  |  | Gum from Akkaraman sheeps | | **Fluorotic sheep**  Ca (mg/g), Mg, Zn, Cu, and Mn μg/g (ppm)  Ca (10; 48.1 ± 5.94 )*, Mg (10; 887 ± 109.84)*, Zn (10; 22.2 ± 3.94), Cu (10; 0.35 ± 0.08), and Mn (10; 3.12 ± 0.52 )*  (N; mean ± SD) | | **Non Fluorotic sheep**  Ca (mg/g), Mg, Zn, Cu, and Mn μg/g (ppm)  Ca (10; 33.5 ± 4.49), Mg (10; 1198 ± 173), Zn (10; 23.8 ± 5.04), Cu (10; 0.32 ± 0.03), and Mn (10; 0.27 ± 0.07)  (N; mean ± SD) | |  |
| Çetin and Yur, 2016 | Fluorotic and non-fluorotic sheep | Muscle | | **Fluorotic sheep**  Fe, Zn, Ni, and Cu (mg/g)  Fe (15; 3.806 ± 0.151), Zn (15; 0.363 ± 0.018)*, Ni (15; 0.089 ± 0.005), and Cu (15; 0.063 ± 0.001)*  (N; mean ± SEM) | | **No fluorotic sheep**  Fe, Zn, Ni, and Cu (mg/g)  Fe (10; 3.53 ± 0.214), Zn (10; 0.526 ± 0.011), Ni (10; 0.088 ± 0.005), and Cu (10; 0.060)  (N; mean ± SEM) | |  |
|  |  | Kidney tissue | | **Fluorotic sheep**  Fe, Zn, Ni, and Cu (mg/g)  Fe (15; 13.4 ± 0.496)*, Zn (15; 0.384 ± 0.02)*, Ni (15; 0.270 ± 0.007)*, and Cu (15; 0.078 ± 0.003)  (N; mean ± SEM) | | **No fluorotic sheep**  Fe, Zn, Ni, and Cu (mg/g)  Fe (10; 8.88 ± 0.11), Zn (10; 0.270 ± 0.007), Ni (10;0.249 ± 0.010), and Cu (10; 0.066 ± 0.0037)  (N; mean ± SEM) | |  |
| Cetin et al., 2020 | Akkaraman sheep with and without fluorosis in the Agri region, to the north of Lake Van, Turkey. | Wool from Akkaraman sheeps | | **Akkaraman sheep with fluorosis in the Agri region, to the north of Lake Van**  Fe, Zn, Ni, Cu, Mn, Co, and Cd (mg/L [ppm])  Fe (15; 0.675 ± 0.352), Zn (15; 2.69 ± 0.611)*, Ni (15; 0.139 ± 0.041), Cu (15; 0.062 ± 0.018)*, Mn (15; 0.333 ± 0.163), Co (15; 0.033 ± 0.018), and Cd (15; 0.007 ± 0.001)  (N; mean ± SD) | | **Akkaraman sheep without fluorosis in the Agri region, to the north of Lake Van**  Fe, Zn, Ni, Cu, Mn, Co, and Cd (mg/L [ppm])  Fe (10; 0.694 ± 0.375), Zn (10; 3.35 ± 0.712), Ni (10; 0.142 ± 0.046), Cu (10; 0.090 ± 0.027), Mn (10; 0.348 ± 0.124), Co (10; 0.040 ± 0.008), and Cd (10; 0.008 ± 0.001)  (N; mean ± SD) | |  |

**Legend:** Al, Aluminum; As, Arsenic; Ca, Calcium; Cd, Cadmium; Co, Cobalt; Cu, Copper; F, Fluoride; Li, Lithium; Mg, Magnesium; Mn, Manganese; Mo, Molybdenum; NaF, Sodium fluoride; Pb, Lead; Sr, Strontium; Zn, Zinc; iAs, inorganic arsenic. MAs, monomethylated arsenic species. DMAs, dimethylated arsenic. This table summarizes the experimental models, exposure routes, target tissues or organs, and individual quantitative results reported in the studies included in this systematic review. Data are presented as reported in the original publications, including sample size (n) and measures of central tendency and variability (mean ± SD, SEM, or SE). Units of measurement are maintained according to the original studies. Statistically significant differences are indicated by symbols, as defined by the respective authors. * Indicates a statistically significant difference compared with the control group (*p* < 0.05). # indicates a statistically significant difference compared with the fluoride-only group or another specified comparator (*p* < 0.05), as reported in the original studies. Due to substantial heterogeneity in experimental designs, animal models, exposure regimens, outcome measures, and reporting formats, the results were synthesized qualitatively.

| **TABLE S2.** Qualitative synthesis of direction and consistency of effects across included studies. | | | | |
| --- | --- | --- | --- | --- |
| **Author, year** | **Pre-clinical model** | **Target tissue/organ** | **Data synthesis** | **Individual outcomes** |
| Kanwar et al., 1981 | Water intake | Liver from Wistar rats | Group Ix control: Zn ↓ Cu ↑ Mn ↓  Group II x control: Zn ↓ Cu ↓ Mn ↓ | In the liver, there was a significant decrease in Mn, Cu, and Zn levels. In the kidneys, Mn levels decreased while Zn levels increased  In the bones, Cu content decreased, while Mn content increased |
|  |  | Kidney from Wistar rats | Group I x control: Zn ↑Cu ↑ Mn ↑  Group II x control: Zn ↑ Cu ↑ Mn ↓ |  |
|  |  | Femur from Wistar rats | Group I x control: Zn ↑ Cu ↑ Mn ↑  Group II x control: Zn ↓ Cu ↓ Mn ↑ |  |
| Larsen et al., 1981 | Intraperitoneal injection | Plasma from Wistar rats | 6-9 days of life  Group I, II, III x Group VII, VIII, IX respectively: Ca ↓  90-9 days of life  Grupo IV (5 minutes, 15 minutes, 60 minutes) x Group X: Ca ↓  Grupo IV (4 hours, 4 days) x Group X: Ca ↑  Group V (5 minutes, 15 minutes, 60 minutes, 4 hours) x Group XI: Ca ↓  Group V (4 days) x Group XI: Ca↑  Group VI (5 minutes, 15 minutes, 60 minutes, 4 hours ) x Group XII: Ca ↓  Group VI (4 days) x Group XII Ca↑ | Fluoride decreases serum Ca levels |
| Singh and Kanwar, 1981 | Water intake | Kidney from Wistar rats | Group I x control: Cu ↑ Mn ↑ Zn ↑  Group II x control: Cu ↑ Mn ↓ Zn ↑ | A significant increase in Mn and Cu levels in bones was observed in the group exposed to 25 ppm of F |
|  |  | Femur from Wistar rats | Group I x control: Cu ↑ Mn ↑ Zn ↑  Group II x control: Cu ↓ Mn ↑ Zn ↓ |  |
| Singh, 1982 | Water intake | Liver from Swiss albino mice | Group I x control: Mn ↑  Groups II x control: Mn ↓  Groups III, IV, and V x control: Mn ↓ | The presence of F decreases Mn levels in the liver at all F doses and increases Mn levels in the femur at all F doses  In the kidney, Mn increases at a dose of 10 ppm, and decreases at doses of 25, 50, 100, and 200 ppm |
|  |  | Kidney from Swiss albino mice | Group I x control: Mn ↑  Group II, and III x control: Mn ↓  Group IV, and V x control: Mn ↓ |  |
|  |  | Femur from Swiss albino mice | Group I x control: Mn ↑  Group II, III, IV, and V x control: Mn ↑ |  |
| Ericsson et al., 1986 | Diet  Balance (Ca 0.43%, P 0.43%, Mg 485 ppm, K 0.48%) or Imbalance (Ca 0.12%, P 0.82%, Mg 98 ppm, K 0.19%) with water intake | Plasma from Sprague-Dawley rats | Group I x V: Ca ↓ Mg ↓  Group II x VI: Ca ↑ Mg ↑  Group III x VII: Ca ↓ Mg ↑ | Plasma Mg was reduced in the group supplemented with Ca and was not influenced by F  Calcium accumulation was more pronounced in the kidneys than in the heart and aorta, and it was lower in the Mg + F supplemented group  The Ca + F group showed a marked renal accumulation of Ca |
|  |  | Aorta from Sprague-Dawley rats | Group I x V: Ca ↑ Mg ↓  Group II x VI: Mg ↑  Group III x VII: Ca ↓ Mg ↑ |  |
|  |  | Heart from Sprague-Dawley rats | Group I x V: Ca ↓ Mg ↑  Group II x VI: Ca ↓ Mg ↓  Group III x VII: Ca ↓ Mg ↑ |  |
|  |  | Kidney from Sprague-Dawley rats | Group I x V: Ca ↓ Mg ↓  Group II x VI: Ca ↑ Mg ↑  Group III x VII: Ca ↓ Mg ↑ |  |
|  |  | Femur from Sprague-Dawley rats | Group I x V: Ca ↑ Mg ↑  Group II X VI: Ca ↓ Mg ↑  Group III x VII: Ca ↓ Mg ↑ |  |
| Cooke et al., 1990 | Water intake | Liver from Wood mice | Group I x IV: Pb ↓ Zn ↓ Cd ↓  Group II x V: Pb ↑ Zn ↓ Cd ↓  Group III x VIII: Pb ↑ Zn ↓ Cd ↑ | F altered Pb concentration in the femur  The combination of F + Pb significantly increased Cd concentration in the kidneys |
|  |  | Kidney from Wood mice | Group I x IV: Pb ↓ Zn ↓ Cd ↓  Group II x V: Pb ↓ Zn ↑ Cd ↑  Group III x VIII: Pb ↑ Zn ↑ Cd ↑ |  |
|  |  | Femur from Wood mice | Group I x IV: Pb ↑ Zn ↑ Cd ↑  Group II x V: Pb ↓ Zn ↑ Cd ↑  Group III x VIII: Pb ↑ Zn ↓ Cd ↑ |  |
| Koskinen-Kainulainen and Tuomist, 1990 | Water gavage | Serum from Wistar rats | Group I x III: Ca ↑ Mg ↓  Group II x IV: Ca ↓ Mg ↓ | There were no significant differences between intervention and comparator groups |
|  |  | Bone from Wistar rats | Group I x III: Ca ↑ Mg ↓  Group II x IV: Ca ↑ Mg ↑ |  |
|  |  | Urine from Wistar rats | Group I x III: Ca ↑ Mg ↓  Group II x IV: Ca ↓ Mg ↓ |  |
|  |  | Feces from Wistar rats | Ca ↑ Mg ↑ |  |
|  |  | Heart from Wistar rats | Group I x III: K ↑ Na ↓ Ca ↑ Mg ↑  Group II x IV: K ↑ Na ↓ Ca ↑ Mg ↑ |  |
| Krasowska and Wlostowski, 1992 | Water intake | Plasma from Wistar rats | Zn ↓ Fe ↑ | Exposure to F decreased Zn concentrations in the testes, plasma, liver, and kidneys. In bones, there was a tendency for an increase in Zn concentration  Fe concentration was not affected in the testes and plasma, but increased in the liver, kidneys, and bones  No changes were observed in Cu and Mn concentrations in the testes, liver, kidneys, and bones |
|  |  | Liver from Wistar rats | Group I x III (6 weeks): Zn ↑ Fe ↓ Cu ↑ Mn ↑  Group I x III (16 weeks): Zn ↓ Fe ↑ Cu ↓ Mn ↓  Group II x III (6 weeks): Zn ↓ Fe ↑ Cu ↓ Mn ↓  Group II x III (16 weeks): Zn ↓ Fe ↑ Cu ↓ Mn ↓ |  |
|  |  | Kidney from Wistar rats | Group I x III (6 weeks): Zn ↑ Fe ↓ Cu ↑ Mn ↓  Group I x III (16 weeks): Zn ↓ Fe ↑ Cu ↓  Group II x III (6 weeks): Zn ↓ Fe ↑ Cu ↓  Group II x III (16 weeks): Zn ↓ Fe ↑ Cu ↓ Mn ↓ |  |
|  |  | Femur from Wistar rats | Group I x III (6 weeks): Zn ↑ Fe ↑  Group I x III (16 weeks): Zn ↑ Fe ↑  Group II x III (6 and 16 weeks): Zn ↑ Fe ↑ |  |
|  |  | Testis from Wistar rats | Group I x III (6 weeks): Zn ↓ Fe ↑ Cu ↓ Mn  Group I x III (16 weeks): Zn ↓ Fe ↑ Cu ↓ Mn ↓  Group II x III (6 weeks): Zn ↓ Fe ↑ Cu ↓ Mn ↓  Group II x III (16 weeks): Zn ↓ Fe ↑ Cu ↓ Mn ↑ |  |
| Yu et al., 1992 | Diet | Plasma from Sprague-Dawley rats | Se ↑ | In the liver, Se was slightly reduced at higher levels of F and Se combined  F reduces Se-induced liver damage |
|  |  | Liver from Sprague-Dawley rats |  |  |
|  |  | Kidney from Sprague-Dawley rats |  |  |
| Tao et al., 2005 | Diet treatments added to the corn-soybean basal diet | Liver from pigs | Group I x IV: Fe ↓ Cu ↓ Zn ↑ Mn ↓  Group II and III x IV: Fe ↓ Cu ↓ Zn ↓ Mn ↓ | F decreases growth rate and alters the retention of Fe, Cu, Zn, and Mn  Altered levels of iron, copper, zinc, and manganese were observed in most tissues in the 100 and 150 mg/kg fluoride groups  This was not observed at a dose of 50 mg/L |
|  |  | Renal cortex from pigs | Group I x IV: Fe ↓ Cu ↓ Zn ↓  Group II and III x IV: Fe ↑ Cu ↓ Zn ↑ Mn ↓ |  |
|  |  | Heart from pigs | Group I x IV: Fe ↑ Cu ↑ Zn ↓ Mn ↑  Group II x IV: Fe ↓ Cu ↓ Zn ↑ Mn ↓  Group III x IV: Fe ↓ Cu ↓ Zn ↓ Mn ↓ |  |
|  |  | Pancreas from pigs | Group I x IV: Fe ↑ Cu ↑ Zn ↓ Mn ↓  Group II x IV: Fe ↓ Cu ↓ Zn ↓ Mn ↓  Group III x IV: Fe ↓ Cu ↓ Zn ↓ Mn ↓ |  |
|  |  | Spleen from pigs | Group I x IV: Fe ↓ Cu ↓ Zn ↑ Mn ↑  Group II x IV: Fe ↓ Cu ↓ Zn ↑ Mn ↓  Group III x IV: Fe ↓ Cu ↓ Zn ↓ Mn ↓ |  |
|  |  | Brain from pigs | Group I x IV: Fe ↑ Cu ↑ Zn ↑ Mn ↑  Group II x IV: Fe ↓ Cu ↓ Zn ↓ Mn ↓  Group III x IV: Fe ↓ Cu ↓ Zn ↓ Mn ↓ |  |
|  |  | Bone from pigs | Group I x IV: Fe ↓ Cu ↓ Zn ↓ Mn ↑  Group II x IV: Fe ↓ Cu ↑ Zn ↓ Mn ↓  Group III x IV: Fe ↓ Cu ↓ Zn ↓ Mn ↓ |  |
|  |  | Serum from pigs | Group I x IV: Fe ↓ Cu ↓ Zn ↓ Mn ↑  Group II x IV: Fe ↓ Cu ↓ Zn ↓ Mn ↓  Group III x IV: Fe ↓ Cu ↓ Zn ↓ Mn ↓ |  |
| Ranjan et al., 2008 | Cattle endemic for fluoride pollution with clinical lesions suggestive of chronic fluoride toxicity and healthy catlle | Blood from cattle | Cu ↓ Zn ↓ Co ↑ Mn ↓ Mg ↑ | Serum levels of Zn, Cu, and Mn are lower in fluorotic cattle, while Co and Mg levels are higher |
|  |  | Serum from cattle | Ca ↓ |  |
| Niu et al., 2009 | Water intake | Blood from Wistar rats | Group I x III: Pb ↑  Group II x III: Pb ↓ | F and Pb, either isolated or combined, decrease learning ability and glutamate levels in the hippocampus. The presence of F did not significantly alter Pb levels in the blood |
| Karademir, 2010 | Water intake | Serum from Swiss  albino mice | Group I x Group V: Co ↑ Mo ↓ Cr ↓ Mn ↓ Li ↓  Group II x Group V: Co ↑ Mo ↓ Cr ↓ Mn ↓ Li ↓  Group III x Group V: Co ↑ Mo ↓ Cr ↓ Mn ↓ Li ↓  Group IV x Group V: Co ↑ Mo ↓ Cr ↓ Mn ↓ Li ↓ | Serum Co levels increased in the presence of F, while Mo, Cr, and Mn levels decreased with the increasing dose of F |
| Sawan et al., 2010 | Water intake | Whole blood from Wistar rats | Pb ↑ | F increases Pb levels in the blood and calcified tissues |
|  |  | Enamel surface from Wistar rats | Pb ↑ |  |
|  |  | Whole dentine from Wistar rats | Pb ↑ |  |
|  |  | Bone surface from Wistar rats | Pb ↑ |  |
|  |  | Whole bone from Wistar rats | Pb ↑ |  |
| Khandare et al., 2011 | Daily orally administration | Urine from New Zealand white rabbits | Ca ↓ Zn ↑ Mg ↓ Cu ↓ | F did not significantly alter the levels of Ca, Mg, Zn, or Cu compared to the control |
|  |  | Serum from New Zealand white rabbits | Group I x III: Ca ↓ Zn ↓ Mg ↑ Cu ↓  Group II x III: Ca ↑ Zn ↓ Mg ↑ Cu ↓ |  |
| Ranjan et al., 2011 | Water intake | Liver from New Zealand white rabbits | Group I x IV: Zn ↓ Cu ↓ Co ↓ Mn ↓  Group II x IV: Zn ↓ Cu ↑ Co ↓ Mn ↓  Group III x IV: Zn ↓ Cu ↓ Co ↓ Mn ↓ | F decreases Zn levels in the liver, kidney, heart, and lung; reduces Cu levels in the muscle, heart, and lung; and lowers Co and Mn concentrations in the liver, kidney, heart, and lung |
|  |  | Kidney from New Zealand white rabbits | Group I x IV: Zn ↓ Cu ↓ Co ↓ Mn ↓  Group II x IV: Zn ↓ Cu ↓ Co ↓ Mn ↓  Group III x IV: Zn ↓ Cu ↑ Co ↑ Mn ↓ |  |
|  |  | Muscle from New Zealand white rabbits | Group I x IV: Zn ↓ Cu ↓ Co ↓ Mn ↓  Group II x IV: Zn ↓ Cu ↓ Co ↓ Mn ↑  Group III x IV: Zn ↓ Cu ↓ Co ↓ Mn ↓ |  |
|  |  | Heart from New Zealand white rabbits | Group I x IV: Zn ↓ Cu ↓ Co ↓ Mn ↓  Group II x IV: Zn ↓ Cu ↓ Co ↓ Mn ↓  Group III x IV: Zn ↓ Cu ↓ Co ↓ |  |
|  |  | Lung from New Zealand white rabbits | Group I x IV: Zn ↓ Cu ↓ Co ↓ Mn ↑  Group II x IV: Zn ↑ Cu ↓ Co ↓ Mn ↓  Group III x IV: Zn ↑ Cu ↓ Co ↑ Mn ↓ |  |
| Chhabra et al., 2012 | In vivo  Cattle of villages of district Muktsar, a brackish water zone, of Punjab state | Plasma from cattle | Ca ↓ Mg ↓ Fe ↑ Cu ↓ Zn ↓ Mn ↑ | Ca, Mg, Cu, and Zn were significantly lower in the plasma of fluorotic cattle, while Fe and Mn were higher |
| Chen et al., 2013 | Water intake | Blood from Sprague–Dawley rats | Group I x III: Cd ↓  Group II x IV: Cd ↑ | Cd levels in the blood of rats co-exposed to F and Cd (F + Cd) were significantly higher than in rats exposed to Cd alone |
|  |  | Urine from Sprague–Dawley rats | Cd ↓ |  |
| Zhang et al., 2013 | Water intake | Blood from Sprague–Dawley rats | Cd ↓ | Synergism (F enhances the effect of Cd)  The level of F in urine was significantly lower during co-exposure with Cd  Co-exposure to Cd and F increases liver and kidney damage compared to exposure to Cd or F alone |
|  |  | Urinary from Sprague–Dawley rats | Cd ↓ |  |
| Aydin et al., 2014 | Akkaraman sheep with and without fluorosis | Bone from Akkaraman sheep | Ca ↑ Cu ↓ Mg ↓ Mn ↑ Zn ↑ | Fluorosis group in bone: the level of Cu decreased, and other minerals were unchanged  Fluorosis group in the teeth: Ca increased, and Mg, Zn, and Mn decreased  In the gums, in the fluorosis group, the Ca and Mn increased, and magnesium decreased |
|  |  | Tooth from Akkaraman sheep | Ca ↑ Cu ↓ Mg ↓ Mn ↓ Zn ↓ |  |
|  |  | Gum from Akkaraman sheep | Ca ↑ Cu ↑ Mg ↓ Mn ↑ Zn ↓ |  |
| Khandare et al., 2015 | Water intake | Serum from Guinea pigs (*Cavia porcellus*) | Group I x III: Sr ↑ (4 months and 6 months) Ca ↓  Group II x IV: Sr ↓ Ca ↑ | Both F and Sr, when isolated, did not alter calcium levels in plasma  However, the combination of F + Sr decreased plasma calcium levels |
| Li et al., 2015 | Water intake | Urine from Wistar rats | Group I x III: Al ↑  Group II x IV: Al ↑ | Both Al and F reduced learning and memory capacity. The presence of F increased Al levels in urine and the brain compared to the control group, and decreased Al levels in the brain compared to the group exposed only to Al |
|  |  | Brain from Wistar rats | Group I x III: Al ↑  Group II x IV: Al ↓ |  |
| Çetin and Yur, 2016 | Fluorotic and no fluorotic sheep | Muscle from sheep | Cu ↑ Zn ↓ Ni ↑ Fe ↑ | In the muscle tissue of sheep with fluorosis, Cu was higher compared to the control, while Zn was higher in the control group  The concentrations of Zn, Fe, and Ni were higher in the liver of sheep with fluorosis, and there was no significant difference in Cu levels |
|  |  | Kidney tissue from sheep | Cu ↑ Zn ↑ Ni ↑ Fe ↑ |  |
| Cetin et al., 2020 | Akkaraman sheep with and without fluorosis in the Agri region, to the north of Lake Van | Wool from Akkaraman sheep | Ni ↓ Cu ↓ Mn ↓ Fe↓ Zn ↓ Cd ↓ Co ↓ | In sheep with fluorosis, Cu and Zn were significantly reduced, while Ni, Mn, Fe, and Co were decreased, but not significantly compared to the control |
| Dey Bhowmi, 2020 | Water intake | Bone from Swiss albino mice | Group I x VI: Ca ↓ Zn ↓ Mg ↑ Fe ↓ Mn ↓ Se ↑ Ni ↓ K ↓  Group II x VI: Ca ↓ Zn ↓ Mg ↑ Fe ↑ Mn ↓ Se ↓ Ni ↓ K ↓  Group III x VI: Ca ↓ Zn ↓ Mg ↑ Fe ↑ Mn ↓ Se ↓ Ni ↓ K ↓ | F significantly decreased Zn, Mn, K, and Ni levels, while increasing Mg and Fe levels in the bone |
| Li et al., 2023 | Water intake | Liver from Sprague-Dawley rats | Group I, II, and III x IV: Cd ↓  Group I, and II x V: Cd ↑  Group III x V: Cd ↑ | After a low supplement of F, Cd levels declined by 31.29 % for liver, 18.31 % for kidney, and 2.89 % for colon, respectively |
|  |  | Kidney from Sprague-Dawley rats | Group I, and II x IV: Cd ↓  Group III x IV: Cd ↓  Group I, II, and III x V: Cd ↑ |  |
|  |  | Colon from Sprague-Dawley rats | Group I, and II x IV: Cd ↓  Group III x IV: Cd ↓  Group I, and II x V: Cd ↑  Group III x V: Cd ↑ |  |
| Pena et al., 2023 | Water intake | Kidney from C57BL/6 mice | Group I x III: iAs ↓ DMAs ↑ Sum As species↑  Group II x IV: iAs ↓ MAs ↓ DMAs↓ Sum As species ↓ | Reduced deposition of As in the presence of F |
|  |  | Liver from C57BL/6 mice | Group I x III: iAs ↓ MAs ↓ DMAs ↑ Sum As species ↑  Group II x IV: iAs ↓ MAs ↓ DMAs ↓ Sum As species ↓ |  |
|  |  | Lung from C57BL/6 mice | Group I x III: MAs ↓ DMAs ↑ Sum As species ↑  Group II x IV: iAs ↓ MAs ↓ DMAs ↓ Sum As species ↓ |  |
|  |  | Urinary bladder from C57BL/6 mice | Group I x III: MAs ↑ DMAs ↑ Sum As species ↑  Group II x IV: iAs ↓ MAs ↓ DMAs ↓ Sum As species ↓ |  |
|  |  | Urine from C57BL/6 mice | Group I x III: iAs ↓ DMAs ↓ Sum As species ↓  Group II x IV: iAs ↑ MAs ↑ DMAs ↓ Sum As species ↓ |  |
| Hu et al., 2024 | Water intake | Bone from wild-type mice | Ca ↓ | Fluoride treatment caused a dramatic increase in F content and marked decreases in Ca content in the bones of the F and FCa groups |
|  |  | Bone from Parkin -/- C57BL/6 mice | Ca ↓ |  |
|  |  | Bone from C57BL/6 mice | Ca ↓ |  |
| Li et al., 2024a | Daily gavage | Testis from IL-17A knockout C57BL/6J mice and wild type (WT) mice | Fe ↑ | The presence of F increases Fe levels in testicular tissue |
| Li et al., 2024b | Water intake | Colon from Sprague-Dawley rats | Group I x IV: Cd ↓  Group II, and III x IV: Cd ↓  Groups I, II, and III x V: Cd ↑ | The Cd levels in colon contents were attenuated by 45.45%, 28.11%, and 19.54 % by F supplement, respectively |
| Sawar et al., 2024 | Water intake | Blood from Wistar rats | Ca ↓ Fe ↓ Cu ↓ Mg ↓ Zn ↓ | In the fluoride-exposed groups, the blood concentrations of essential minerals were significantly lower than the control  In the stool of fluoride-treated groups, the concentrations of these compounds increased with increasing fluoride dosage |
|  |  | Stool from Wistar rats | Group I x control: Ca ↑ Fe ↑ Cu ↑ Mg ↑ Zn ↑  Group II x control: ↑ Ca ↑ Fe ↑ Cu ↑ Mg ↑ Zn ↑ |  |

**Legend:** Al, Aluminum; As, Arsenic; Ca, Calcium; Cd, Cadmium; Co, Cobalt; Cu, Copper; F, Fluoride; Li, Lithium; Mg, Magnesium; Mn, Manganese; Mo, Molybdenum; NaF, Sodium fluoride; Pb, Lead; Sr, Strontium; Zn, Zinc; iAs, inorganic arsenic. MAs, monomethylated arsenic species. DMAs, dimethylated arsenic. ↑ increase in concentration; ↓ decrease in concentration. Statistically significant differences reported in the original articles are indicated in blue.
